# Boldness in Male and Female Zebrafish (*Danio rerio*) Is Dependent on Strain and Test

**DOI:** 10.3389/fnbeh.2019.00248

**Published:** 2019-11-19

**Authors:** Arshi Mustafa, Erika Roman, Svante Winberg

**Affiliations:** ^1^Department of Neuroscience, Behavioral Neuroendocrinology Group, Uppsala University, Uppsala, Sweden; ^2^Department of Organismal Biology, Uppsala University, Uppsala, Sweden; ^3^Department of Pharmaceutical Biosciences, Division of Pharmacology, Neuropharmacology, Addiction and Behavior Unit, Uppsala University, Uppsala, Sweden; ^4^Department of Anatomy, Physiology and Biochemistry, Division of Anatomy and Physiology, Swedish University of Agricultural Sciences, Uppsala, Sweden

**Keywords:** shelter test, novel tank diving test, scototaxis test, light/dark preference test, AB, wild-type, spiegeldanio, fibroblast growth factor receptor 1a

## Abstract

Differences in selection pressure in nature and labs have profound effects on zebrafish strains. The widely used AB strain of zebrafish has been domesticated over several decades. Recently, there has been an upsurge in the availability of genetically modified lines, e.g., the spiegeldanio (*spd*), which has a mutation in the fibroblast growth factor receptor 1a (*fgfr1a*) gene. This mutant strain (*fgfr1a*) has previously been reported to be bolder than fish of the Tübingen strain, from which it was generated. Our knowledge on behavioral differences between different zebrafish strains, relative to wild-caught zebrafish, is limited. In the present study we compare behaviors related to interpretation of boldness in male and female offspring (F1) of wild-caught fish, AB and *fgfr1a*^−/−^ zebrafish. A second aim of the study was to compare the behavior of fish from these strains when tested in different behavioral assays, i.e., shelter seeking, novel tank diving and scototaxis tests. The results demonstrate that behavioral variation exists both within and between the strains, but interpretation of boldness reveals a complex pattern in which behavior differs between strains but is also related to sex and test. Therefore, a careful assessment of various strains of fish using both males and females is warranted in order to strengthen interpretation of results. This is especially important in studies where zebrafish are used as model organisms for human conditions as well as studies evaluating the effects of pharmacological or toxicological substances on behavior.

## Introduction

The zebrafish is a popular model organism in biomedical research, and the number of strains and lines available are increasing (Kalueff et al., 2014; Stewart et al., 2014; Maximino et al., 2015). In nature, zebrafish occurs in diverse habitats ranging from small ponds and rice paddies to larger streams (Spence et al., 2008). Thus, not surprisingly, large inter-population differences in behavior have been reported (Roy and Bhat, 2018a). Moreover, the captive environment, which is highly stable and where threats like predators are lacking, is known to result in rapid domestication with large behavioral effects, such as increased boldness (Huntingford, 2004; Huntingford and Adams, 2005; Agnvall et al., 2015). Boldness and shyness mainly refer to the willingness among individuals to take risks, especially in novel environments. Bold individuals are explorative and risk taking while shy individuals are characterized by low exploration and a passive response (Sih et al., 2004).

Different domesticated zebrafish strains are likely to differ in behavior as a result of differences in origin as well as in the level of domestication and inbreeding. Our knowledge on behavioral differences between different zebrafish strains is limited. Still, such knowledge is important since the use of different strains makes comparisons of results from different labs difficult. The AB fish is one commonly used domesticated strain, and recently there has been an upsurge in genetically modified fish. One such example is the spiegeldanio (*spd*) line, which has a mutation in the fibroblast growth factor receptor 1a (*fgfr1a*) gene (Norton et al., 2011). Norton et al. (2011) showed that the *fgfr1a*^−/−^ fish were bolder than wild-type fish of the Tübingen strain, another highly domesticated zebrafish strain, from which the *fgfr1a*^−/−^ fish was generated. Moreover, due to domestication effects, the AB strain has been reported to be bolder and more explorative than wild-type fish (Wright et al., 2006). Thus, it is of importance to compare boldness of AB and *fgfr1a*^−/−^ zebrafish and to compare the domesticated strains to wild-caught fish using both males and females.

The role of sex on personality in zebrafish has been documented but the reports are conflicting (Genario et al., 2019). In wild-caught fish, males were found to be bolder than females (Roy and Bhat, 2018a). This finding agrees with that of Dahlbom et al. (2011) who also showed that males are bolder than females. However, other studies (Moretz et al., 2007; Conrad et al., 2011; Norton and Bally-Cuif, 2012) report that females are bolder than males. These latter studies used domesticated versions of wild-caught fish whereas Dahlbom et al. (2011) used wild-caught fish that had been reared in the lab for 15 months. These contrasting conditions make it difficult to draw conclusions about sex differences in boldness.

Common tests used for behavioral characterization of adult zebrafish include the novel tank diving, open field and scototaxis tests (Collier et al., 2017; Kysil et al., 2017). However, there is no consensus as to what test to use in order to characterize zebrafish as bold and shy, respectively (Moretz et al., 2007; Conrad et al., 2011; Norton and Bally-Cuif, 2012), and it is likely that results can vary depending on the test chosen. In the present experiment, males and females of domesticated AB fish, genetically modified *fgfr1a*^−/−^ fish and offspring (F1) of wild-caught zebrafish were compared in three commonly used behavioral tests, i.e., the shelter, novel tank diving and scototaxis tests to address if there were: (1) sex differences in boldness within the respective strains; and (2) strain differences in boldness within the respective sex.

## Materials and Methods

### Zebrafish

This study comprised a total of 54 zebrafish; nine male and nine female offspring of wild-caught zebrafish (F1), nine male and nine female of AB and nine male and nine female *fgfr1a*^−/−^ zebrafish. The wild-caught fish were collected from eight different pond locations in India (courtesy Prof. Allan V. Kalueff, Southwest University, China) and the F1 generation, hereafter referred to as wild, was used in the present experiment. The AB line was obtained from the SciLife Lab facility at Uppsala University, Uppsala, Sweden^1^. The *fgfr1a*^−/−^ fish used were the offspring of *fgfr1a*^−/−^ fish, homozygote for the *fgfr1a* t3R705H allele (Rohner et al., 2009), obtained from the lab of Prof. Darren Gilmour of the European Molecular Biology Laboratory, Heidelberg, Germany. All the fish were adults at the time for the experiment and of similar body mass, length and age.

The fish were housed in 9.5 L trapezoidal tanks at 27–28°C in an Aquaneering zebrafish rearing system at Uppsala University Biomedical Centre. The animals were kept on a 14L:10D photoperiod with lights on at 07:00 am. Fish tanks were supplied with recirculating Uppsala municipal tap water of which 10% was exchanged daily. Fish were fed twice daily with tropical energy food (Aquatic Nature, Belgium) and Artemia (Platinum Grade 0, Argentemia, Argent, Aquaculture, Redmond, USA).

The use of animals was approved by the Uppsala Regional Animal Ethical Committee (permit C55/13) and followed the guidelines of the Swedish Legislation on Animal Experimentation (Animal Welfare Act SFS1998:56) and the European Union Directive on the Protection of Animals Used for Scientific Purposes (Directive 2010/63/EU).

### Experimental Design

Male and female wild, AB and *fgfr1a*^−/−^ fish were tested in the shelter, the novel tank diving and the scototaxis tests with a duration of 1 week in between the respective test. The fish to be tested was netted out from the home tank and placed in the respective arenas. The test arenas were placed on an infrared (IR) light board, and the behavior of the fish was recorded from above using an IR sensitive camera (TK-C9510E, JVC, UK). In the shelter test, three fish were tested in parallel, in the novel tank diving test two fish were tested in parallel and in the scototaxis test one fish was tested at a time. The behavior of each individual fish was tracked using Ethovision^®^ XT 12.0 (Noldus Information Technology, Wageningen, Netherlands). In each test the latency (s) to first visiting the zones, frequency of visits, duration (s) in the different zones, duration per visit (s) to the different zones, duration moving (s), duration (s) of immobility, total distance (cm) moved as well as the mean velocity (cm/s) in all zones were registered. Moreover, the duration (s) moving, duration (s) of immobility, total distance (cm) moved as well as the mean velocity (cm/s) in the arena was registered and the total activity (sum of all frequencies) was calculated. All the tests were performed in the day time (between 09:00 am and 04:00 pm). In between tests, the fish were returned to their home tanks.

#### Shelter Test

This test was similar to the shelter test described by Dahlbom et al. (2011). A circular arena of dimensions 19 × 8 cm (diameter × height) made up of poly methyl methacrylate plastic was used. The wall of the arena was opaque, which prohibited the transfer of any visual information. The arena was filled to a depth of 3.5 cm with Uppsala municipal water maintained at 27–28°C. After placing the fish inside the arena, one half of the arena was covered with a transparent lid and the other half was covered with a black lid (IR transparent), resulting in an open and a sheltered half. The fish was allowed to explore the arena for 14 min. Behavior was registered in the open and the sheltered zones, respectively.

#### Novel Tank Diving Test

A clear cuboidal tank of dimension 25 × 20 × 5 cm (length × height × width) was used (Kysil et al., 2017). The arena was filled to a depth of 15 cm with Uppsala municipal water maintained at 27–28°C. The fish was allowed to explore the tank for 6 min. For analysis of behavior, the tank was divided into three zones of equal size (3 cm); bottom, middle and top zone.

#### Scototaxis Test

A cuboidal scototaxis tank of dimension 45 × 15 × 15 cm (length × height × width) was used (Kysil et al., 2017). The whole apparatus was made up of poly methyl methacrylate plastic. The tank was divided into three compartments with two analogous end compartments (dimensions 22.5 × 15 × 15 cm), one of which was black and one of which was white. A smaller central compartment (dimension 10 × 15 × 15 cm) represented the intersection between the black and white compartments and therefore was half black and half white in composition. The arena was filled to a depth of 6 cm with Uppsala municipal water maintained at 27–28°C.

The fish was released in the central compartment and allowed to habituate for 3 min. Following habituation, the central compartment was gently removed and the fish was allowed to freely explore the arena for 14 min. Behavior was registered in the black and the white compartments, respectively.

### Statistical Analyses

One AB male zebrafish was lost in the scototaxis test due to technical difficulties with the recording. Statistical analyses were carried out in Statistica 13.2 (Dell Inc., Tulsa, OK, USA). If a zone was not visited, this was considered a missing value in the statistical analysis. The descriptive parameters did not show a normal distribution according to the Shapiro–Wilk’s W test and consequently, non-parametric statistics were used. Sex differences within the respective strain were investigated using the Mann–Whitney *U*-test with continuity correction. Strain differences within the respective sex were analyzed using the Kruskal–Wallis ANOVA by ranks followed by the Mann–Whitney *U*-test with continuity correction when appropriate. Finally, for activity measures, i.e., total activity, total distance and mean velocity, the total trial time was divided into 2-min bins for analysis of activity over time. The non-parametric Friedman test was used to analyze within strain differences over time. If significant, the Wilcoxon signed-rank test was used to compare the first and the last time point. Data were considered statistically significant at *p* < 0.05.

In addition to conventional statistical analyses, partial least squares to latent structures discriminant analysis (PLS-DA) was used in order to visualize the potential separation of groups and behavioral parameters of relevance for such separations. Multivariate data analyses were performed using SIMCA-P + 15.0 (Umetrics, Sweden).

## Results

### Shelter Test

**Figure 1:**
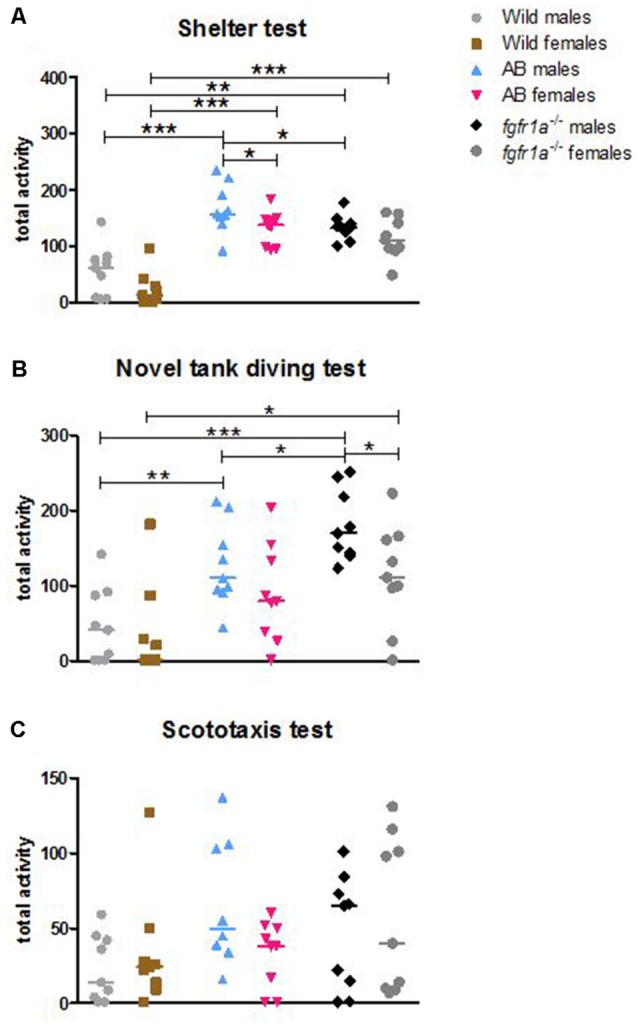
Total activity, i.e., sum of all frequencies, in the shelter **(A)**, novel tank diving **(B)** and scototaxis **(C)** tests in male and female adult offspring of wild-caught (wild), AB and *fgfr1a*^−/−^ zebrafish. Values represent individual fish and the line marks the group median. *p < 0.05, **p < 0.01, ***p < 0.001 (Mann–Whitney *U*-test).

The descriptive results from the 14-min trial in the shelter test are shown in Supplementary Table S1. In offspring of wild-caught zebrafish (wild), minor differences between males and females were observed; wild males swam for longer distance under the shelter and also made more transitions between the sheltered and open area of the arena (Supplementary Table S1), as compared to females. In the AB fish, males made more visits to the open area and had higher velocity there than AB females. In the sheltered area, AB males had higher frequency of visits relative to female AB fish (Supplementary Table S1) and AB males were more active in the arena than AB females with higher total activity (Figure 1A, Supplementary Table S1). In the *fgfr1a*^−/−^ fish, sex-based differences were found in the sheltered area, where *fgfr1a*^−/−^ males swam for longer distance had higher velocity and moved for longer duration than *fgfr1a*^−/−^ females. Overall, in the arena, the *fgfr1a*^−/−^ males swam for longer distance and also had higher velocity than *fgfr1a*^−/−^ females (Supplementary Table S1).

The descriptive results from the 14-min trial in the shelter test revealed differences between male fish (Supplementary Table S1, statistics for strain comparisons are shown in Supplementary Table S2). In the open area, AB and *fgfr1a*^−/−^ males had shorter latency to first visit, made more visits and spent more time moving as compared to the wild fish. Moreover, AB males had higher velocity than wild males. Finally, *fgfr1a*^−/−^ males had a lower frequency of visits in the open area relative to AB males (Supplementary Tables S1, S2). In the sheltered area, AB and *fgfr1a*^−/−^ males made more visits, had a shorter duration per visit, moved a longer distance and had a higher velocity than wild males. Moreover, AB and *fgfr1a*^−/−^ males spent a longer time moving and less time in immobility relative to wild males. Finally, male *fgfr1a*^−/−^ fish made fewer visits compared to AB males (Supplementary Tables S1, S2). With regard to activity in the arena, AB and *fgfr1a*^−/−^ males had higher total activity (Figure 1A, Supplementary Table S1), moved for a longer distance, had higher velocity, longer duration moving and less time in immobility, and made more zone transitions compared to wild males (Supplementary Tables S1, S2). Finally, male *fgfr1a*^−/−^ fish had lower total activity relative to AB males (Figure 1A, Supplementary Tables S1, S2).

The descriptive results from the 14-min trial in the shelter test revealed differences between female fish (Supplementary Tables S1, S2). In the open area, AB and *fgfr1a*^−/−^ females made more visits, swam for a longer distance and spent longer time moving than wild females. There was no significant difference between AB and *fgfr1a*^−/−^ females. In the sheltered area, AB and *fgfr1a*^−/−^ females made more visits, spent less time per visit, moved a longer distance, had a higher velocity, and spent longer time moving and less time in immobility than the wild females. No difference between AB and *fgfr1a*^−/−^ females was revealed (Supplementary Tables S1, S2). With regard to activity in the arena, AB and *fgfr1a*^−/−^ females had higher total activity (Figure 1A, Supplementary Tables S1, S2), swam for a longer distance, had higher velocity, spent longer time moving, displayed less time in immobility and made more zone transitions (Supplementary Tables S1, S2) than wild females. Overall, no difference in activity was found between AB and *fgfr1a*^−/−^ fish (Supplementary Tables S1, S2).

**Figure 2:**
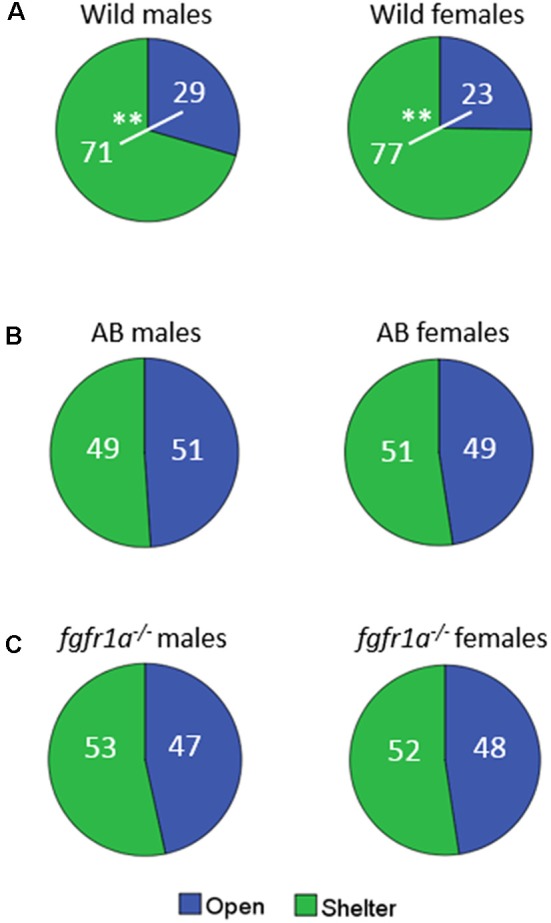
Percentage duration of time spent in the open and sheltered area, respectively, in the shelter test in male and female adult offspring of wild-caught (wild, **A**), AB **(B)** and *fgfr1a*^−/−^ **(C)** zebrafish. Values represent group mean in the respective zone. **p < 0.01 for significant differences in time spent in open and shelter zones within the respective group (Mann–Whitney *U*-test).

The distribution of time spent in the open and sheltered areas respectively is shown in Figure 2. Both wild males and females spent significantly more time under the shelter than in the open area of the arena (Figure 2A), while no significant difference was observed for male and female AB or *fgfr1a*^−/−^ fish (Figures 2B,C).

In summary, sex differences were found within the AB and *fgfr1a*^−/−^ strains, respectively, and within the respective sex the AB and *fgfr1a*^−/−^ fish differed from the wild fish. This pattern is supported by the PLS-DA (Supplementary Figure S1) in which the first component was significant (*R*^2^*X* = 0.662, *R*^2^*Y* = 0.145, Q^2^ = 0.131). AB and *fgfr1a*^−/−^ fish are located in the left quadrants and clearly separated from the wild-caught fish, which were characterized by higher immobility and time spent in the shelter.

#### Activity Over Time in the Shelter Test

**Figure 3:**
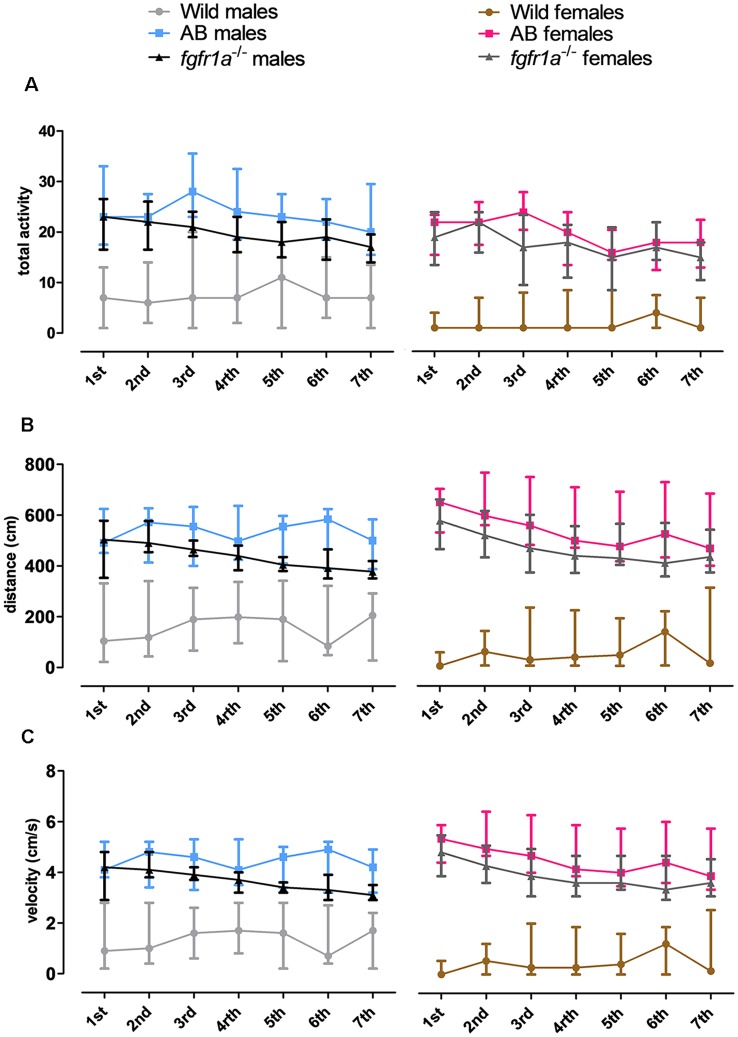
The 14-min trial in the shelter test divided into seven 2-min time bins for total activity, i.e., sum of all frequencies **(A)**, total distance traveled **(B)** and mean velocity **(C)** in the arena in male and female adult offspring of wild-caught (wild), AB and *fgfr1a*^−/−^ zebrafish. Data are presented as median and quartile range. No difference between the first and the last time bin was revealed within the respective groups (Wilcoxon signed-rank test).

The 14-min trial in the shelter test was divided into seven 2-min time bins for total activity, total distance traveled and mean velocity in order to investigate within strain differences over time (Figure 3). In both males and females, wild fish had consistently lower activity relative to AB and *fgfr1a*^−/−^ fish. In males, no within strain differences in behavior over time were observed in wild and AB fish for any parameter (Figure 3). In *fgfr1a*^−/−^ males, there was an overall effect for distance moved ($\chi_{\left(6\right)}^{2}$ = 19.2, *p* = 0.004) and velocity ($\chi_{\left(6\right)}^{2}$ = 19.2, *p* = 0.004), even though the *post hoc* analysis did not reveal any difference between the first and the last time bin (Figures 3B,C). In females, no within strain differences in behavior over time were observed in wild fish (Figure 3). In AB females, there was no significant effect on overall activity over time. However, there was a significant effect on overall distance moved ($\chi_{\left(6\right)}^{2}$ = 17.7, *p* = 0.007) and velocity ($\chi_{\left(6\right)}^{2}$ = 17.7, *p* = 0.007). Still, comparisons between the first and the last time bin revealed no significant difference in either distance moved or velocity (Figures 3B,C). In *fgfr1a*^−/−^ females, there was an overall effect for activity ($\chi_{\left(6\right)}^{2}$ = 14.7, *p* = 0.022), distance moved ($\chi_{\left(6\right)}^{2}$ = 26.8, *p* = 0.0002) and velocity ($\chi_{\left(6\right)}^{2}$ = 26.8, *p* = 0.0002). However, comparisons between the first and the last time bin revealed no significant difference in any of the parameters (Figure 3).

### Novel Tank Diving Test

The descriptive results from the 6-min trial in the novel tank diving test are shown in Supplementary Table S3. In offspring of wild-caught zebrafish, no differences between males and females were revealed, except that males that were visiting the top zone had higher velocity than females (Supplementary Table S3). In the AB fish, the only difference between males and females was that AB males made more visits to and spent less time per visit in the bottom zone than AB females (Supplementary Table S3). In the *fgfr1a*^−/−^ fish, pronounced sex differences were found. In the bottom zone, males spent shorter time and time per visit, swam a shorter distance and spent shorter time moving and less time immobile compared to the females (Supplementary Table S3). Moreover, in the top zone the males made more visits, spent longer time, swam a longer distance, and had longer duration moving and more time in immobility relative to the females (Supplementary Table S3). Finally, *fgfr1a*^−/−^ males had higher total activity in the arena (Supplementary Table S3, Figure 1B) and made more zone transitions (middle to top and top to middle) than female *fgfr1a*^−/−^ fish (Supplementary Table S3).

The descriptive results from the 6-min trial revealed differences between male fish, most notably related to the bottom and top zones, respectively (Supplementary Table S3, statistics for strain comparisons are shown in Supplementary Table S4). In the bottom zone, AB and *fgfr1a*^−/−^ males made more visits but had shorter duration and duration per visit compared to wild males. Moreover, AB and *fgfr1a*^−/−^ males spent less time immobile than the wild males (Supplementary Tables S3, S4). In the top zone, *fgfr1a*^−/−^ males made more visits, spent longer time and traveled a longer distance than the wild males, and a similar non-significant tendency was observed when comparing AB to wild males (Supplementary Tables S3, S4). Among AB and *fgfr1a*^−/−^ males, the *fgfr1a*^−/−^ males made more visits, spent longer time, moved a longer distance and spent longer time moving in the top zone relative to AB fish (Supplementary Tables S3, S4). Finally, AB and *fgfr1a*^−/−^ males were more active in the arena as reflected by higher total activity (Supplementary Tables S3, S4, Figure 1B) and spent longer duration moving and less time in immobility than the wild males. Among AB and *fgfr1a*^−/−^ males, *fgfr1a*^−/−^ males made more zone transitions relative to AB males (Supplementary Tables S3, S4).

The descriptive results from the 6-min trial revealed differences between female fish (Supplementary Tables S3, S4). In the bottom zone, AB and *fgfr1a*^−/−^ females had higher velocity and spent less time immobile relative to wild females. Moreover, in this zone, the AB females spent shorter duration per visit compared to wild females (Supplementary Tables S3, S4). In the top zone, more female AB and *fgfr1a*^−/−^ than wild fish visited this zone (Supplementary Table S3). Finally, *fgfr1a*^−/−^ females had higher total activity than the wild females (Supplementary Tables S3, S4, Figure 1B), and AB and *fgfr1a*^−/−^ females traveled a longer distance, had higher velocity, and spent longer duration moving and less time in immobility in the arena than wild females (Supplementary Tables S3, S4).

**Figure 4:**
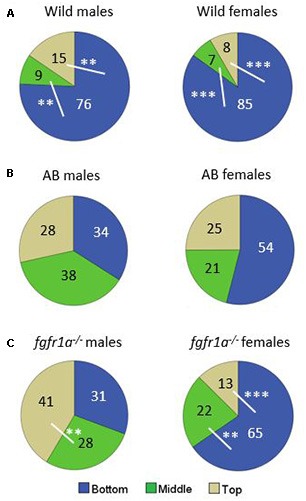
Percentage duration of time spent in the bottom, middle and top zone, respectively, in the novel tank diving test in male and female adult offspring of wild-caught (wild, **A**), AB **(B)** and *fgfr1a*^−/−^ **(C)** zebrafish. Values represent group mean in the respective zone. The white lines represent the zones that are being compared in the statistical analysis. **p < 0.01, ***p < 0.001 for significant differences in time spent in the different zones within the respective group (Mann–Whitney *U*-test).

The distribution of time spent in the different zones is shown in Figure 4. In the wild fish, both males and females spent more time at the bottom of the arena as compared to the middle and the top zones, respectively (Figure 4A). AB males and females spent equal amount of time in the three zones (Figure 4B). The *fgfr1a*^−/−^ males spent significantly more time in the top zone as compared to the middle zone (Figure 4C). By contrast, *fgfr1a*^−/−^ females spent significantly more time in the bottom zone than in the middle and top zones (Figure 4C).

In summary, pronounced sex differences were found within the *fgfr1a*^−/−^ line, and AB and *fgfr1a*^−/−^ fish differed from the wild fish due to the fact that fewer wild fish visited the top zone. This pattern is supported by the PLS-DA (Supplementary Figure S2) in which three components were significant (*R*^2^*X*_(cum)_ = 0.713, *R*^2^*Y*_(cum)_ = 0.213, *Q*^2^_(cum)_ = 0.0861). AB and *fgfr1a*^−/−^ fish are located in the left quadrants, and clearly separated from the wild fish, which were characterized by higher immobility and higher activity in the bottom zone. The sex differences found using conventional statistics are supported by the separation between male and female *fgfr1a*^−/−^ fish (Supplementary Figure S2).

#### Activity Over Time in the Novel Tank Diving Test

**Figure 5:**
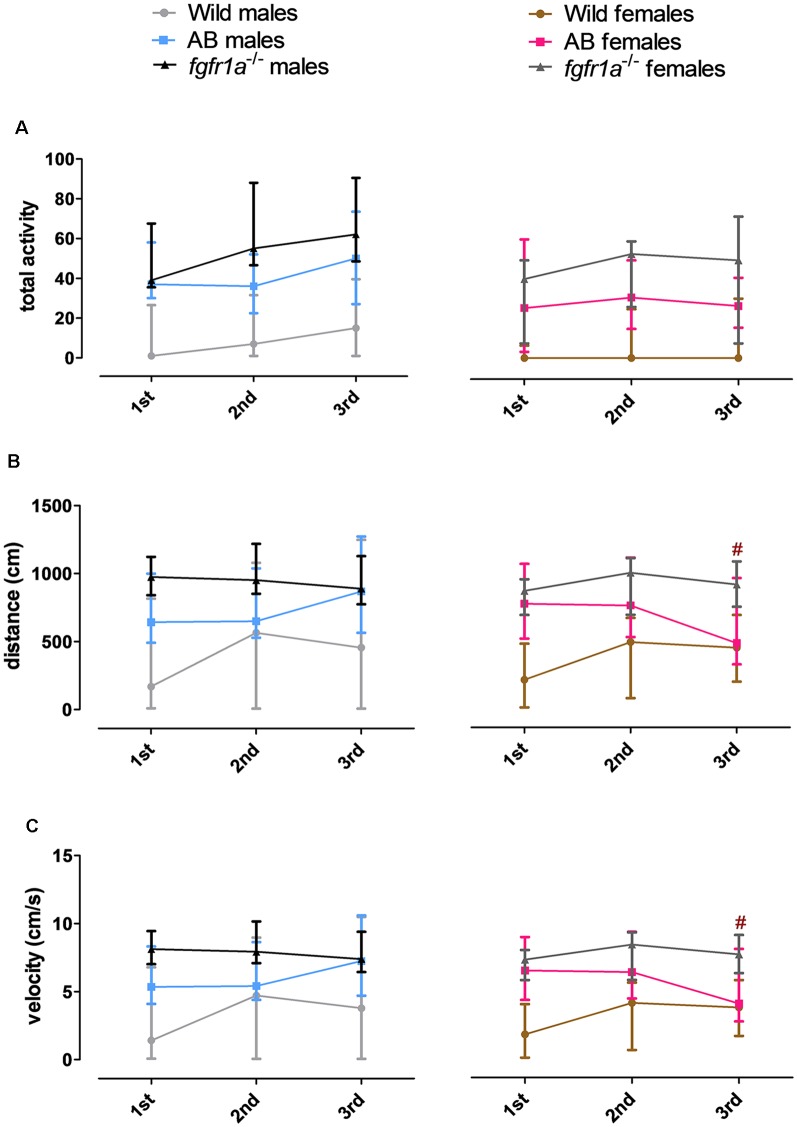
The 6-min trial in the novel tank diving test divided into three 2-min time bins for total activity, i.e., sum of all frequencies **(A)**, total distance traveled **(B)** and mean velocity **(C)** in the arena in male and female adult offspring of wild-caught (wild), AB and *fgfr1a*^−/−^ zebrafish. Data are presented as median and quartile range. ^#^*p* < 0.05 compared to the first time bin within the respective group (Wilcoxon signed-rank test).

The 6-min trial in the novel tank diving test was divided into three 2-min time bins for total activity, total distance traveled and mean velocity in order to investigate within strain differences over time (Figure 5). In both males and females, wild fish had consistently lower total activity relative to AB and *fgfr1a*^−/−^ fish. In males, no difference in behavior across time was revealed in any of the groups (Figure 5). In AB females, an overall difference in distance traveled ($\chi_{\left(2\right)}^{2}$ = 6.0, *p* = 0.050) and velocity ($\chi_{\left(2\right)}^{2}$ = 6.0, *p* = 0.050) over time was observed, with shorter distance traveled and lower velocity during the last time bin relative to the first (Figures 5B,C). A contrasting pattern was observed in the wild females where overall differences in distance traveled ($\chi_{\left(2\right)}^{2}$ = 6.8, *p* = 0.034) and velocity ($\chi_{\left(2\right)}^{2}$ = 6.8, *p* = 0.034) were revealed, but with a non-significant tendency for longer distance traveled and higher velocity during the last time bin relative to the first (Figures 5B,C).

### Scototaxis Test

The descriptive results from the 14-min trial in the scototaxis test are shown in Supplementary Table S5. No sex difference was observed in any of the strains, except that *fgfr1a*^−/−^ females moved significantly less in the white compartment as compared to *fgfr1a*^−/−^ males (Supplementary Table S5).

The descriptive results from the 14-min trial revealed only minor differences between male fish (Supplementary Table S5, statistics for strain comparisons are shown in Supplementary Table S6). In the white compartment, AB and *fgfr1a*^−/−^ males swam longer distance, and had longer duration moving as compared to wild males. Moreover, the *fgfr1a*^−/−^ males spent less time in immobility compared to the wild males (Supplementary Tables S5, S6). In the black compartment, no differences between the males were observed (Supplementary Tables S5, S6). In the total arena, the AB and *fgfr1a*^−/−^ males spent more time moving and less time in immobility relative to the wild males. The *fgfr1a*^−/−^ males also traveled a longer distance in the arena with higher velocity compared to the wild males (Supplementary Tables S5, S6). There were no significant differences in total acitivity between strains (Figure 1C, Supplementary Tables S5, S6).

In agreement with the males, the descriptive results from the 14-min trial revealed only minor differences between the female fish (Supplementary Tables S5, S6). In the white compartment, AB females spent longer duration moving as compared to wild and *fgfr1a*^−/−^ females. In addition, AB and *fgfr1a*^−/−^ females spent less time in immobility in the black compartment as compared to wild females (Supplementary Tables S5, S6). With regard to activity in the arena, AB and *fgfr1a*^−/−^ females spent longer duration moving and less time in immobility relative to wild females (Supplementary Tables S5, S6). Females showed no significant differences in total activity (Figure 1C, Supplementary Tables S5, S6).

**Figure 6:**
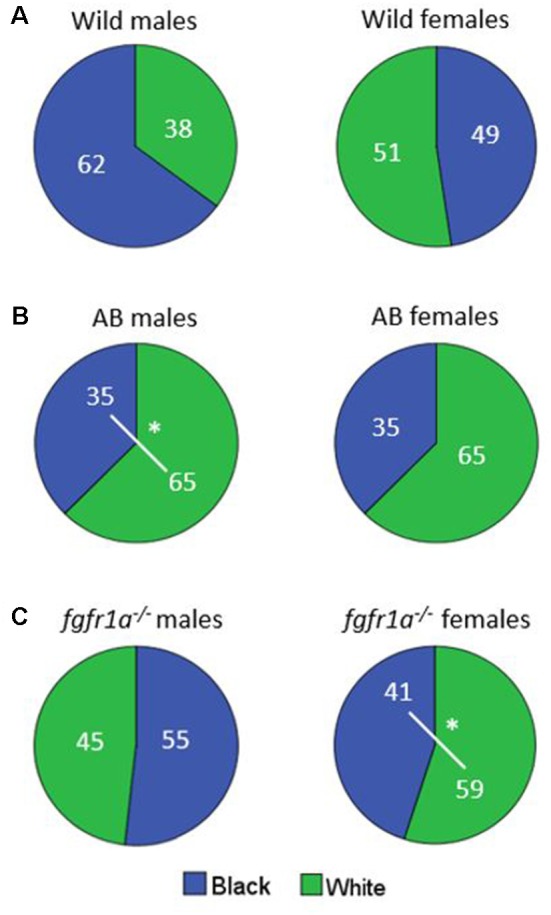
Percentage duration of time spent in the white and black compartment, respectively, in the scototaxis test in male and female adult offspring of wild-caught (wild, **A**), AB **(B)** and *fgfr1a*^−/−^ **(C)** zebrafish. Values represent group mean in the respective zone. ^#^*p* < 0.05 for significant differences in time spent in the different zones within the respective group (Mann–Whitney *U*-test).

The distribution of time spent in the different zones is shown in Figure 6. No differences were revealed in the wild males and females (Figure 6A). AB males spent more time in the white than in the black compartment, with a similar non-significant tendency also for AB females (Figure 6B). The *fgfr1a*^−/−^ males spent approximately equal time in both compartments, while *fgfr1a*^−/−^ females spent more time in the white compartment (Figure 6C).

In summary, no evident sex differences were found nor were there any marked differences between strains either in males or in females. This pattern is further supported by the PLS-DA (Supplementary Figure S3) in which no significant component was generated (first component *R*^2^*X* = 0.412, *R*^2^*Y* = 0.072, Q^2^ = 0.0348, second component *R*^2^*X* = 0.115, *R*^2^*Y* = 0.0599, *Q*^2^ = −0.00125). Still, the wild fish are separated from the AB and *fgfr1a*^−/−^ and, consistent with the shelter and novel tank diving tests, characterized by increased immobility (Supplementary Figure S3).

#### Activity Over Time in the Scototaxis Test

**Figure 7:**
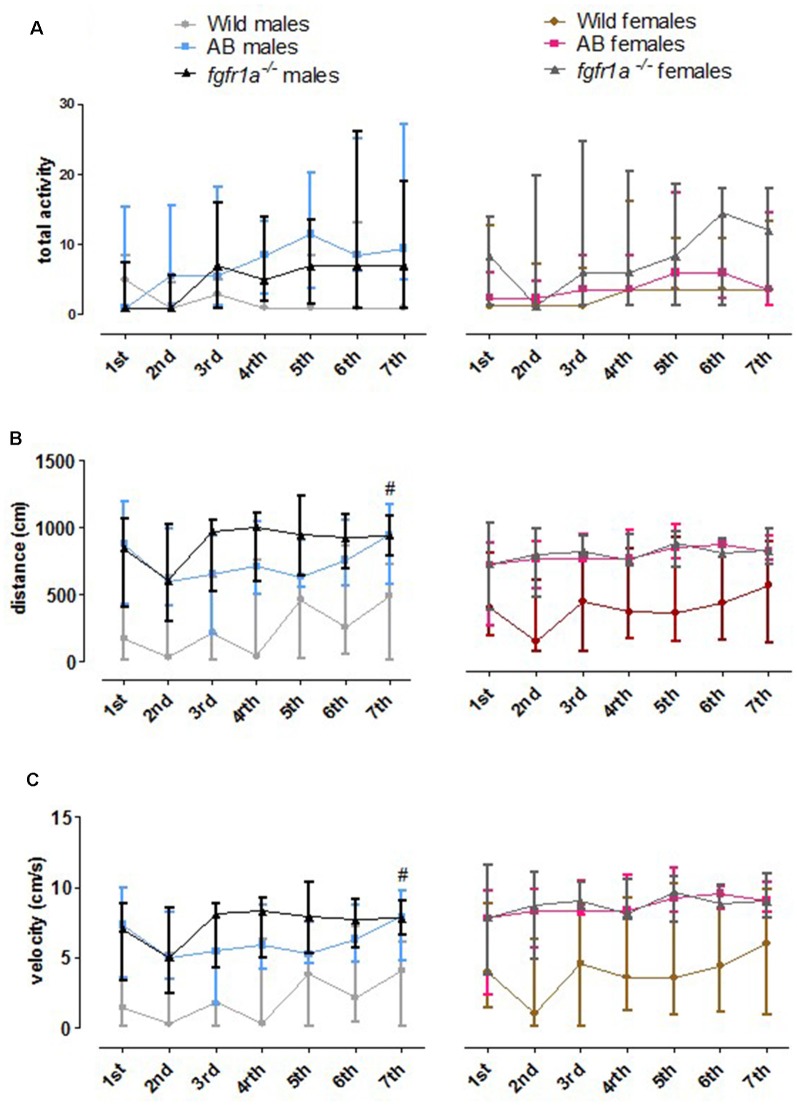
The 14-min trial in the scototaxis test divided into seven 2-min time bins for total activity, i.e., sum of all frequencies **(A)**, total distance traveled **(B)** and mean velocity **(C)** in the arena in male and female adult offspring of wild-caught (wild), AB and *fgfr1a*^−/−^ zebrafish. Data are presented as median and quartile range. ^#^*p* < 0.05 compared to the first time bin within the respective group (Wilcoxon signed-rank test).

The 14-min trial in the scototaxis test was divided into seven 2-min time bins for total activity, total distance traveled and mean velocity in order to investigate within strain differences over time (Figure 7). In wild fish, males and females consistently moved a shorter distance with a lower velocity relative to AB and *fgfr1a*^−/−^ fish. In *fgfr1a*^−/−^ males, overall differences were found for total activity ($\chi_{\left(6\right)}^{2}$ = 12.8, *p* = 0.047), distance moved ($\chi_{\left(6\right)}^{2}$ = 15.7, *p* = 0.016) and velocity ($\chi_{\left(6\right)}^{2}$ = 15.7, *p* = 0.016), with longer distance moved and higher velocity during the last time bin relative to the first (Figures 7B,C). In females, no overall differences were found for any parameter (Figure 7).

### Comparison Between Tests

**Figure 8:**
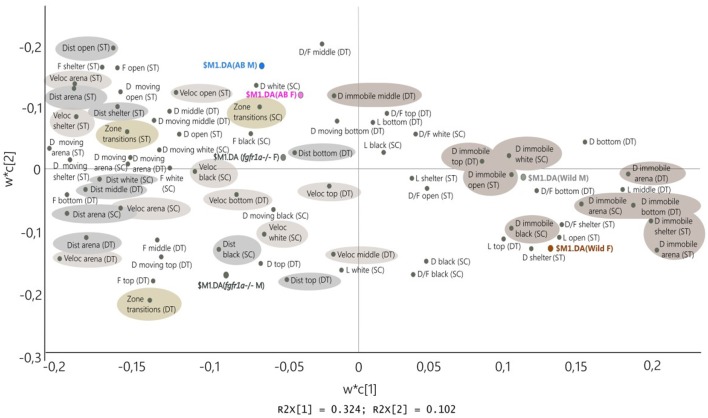
Scatter plot of variable loadings from the PLS-DA (first significant component *R*^2^*X* = 0.324, *R*^2^*Y* = 0.154, Q^2^ = 0.136, second non-significant component *R*^2^*X* = 0.102, *R*^2^*Y* = 0.0716, Q^2^ = 0.011) combining the descriptive parameters from the shelter test (ST), novel tank diving test (DT) and scototaxis test (SC) in male (M) and female (F) adult offspring of wild-caught (wild), AB and *fgfr1a*^−/−^ zebrafish. Abbreviations: D, duration (s); D/F, duration per visit (s); Dist, total distance (cm); DT, novel tank diving test; F, frequency; L, latency (s); SC, scototaxis test; ST, shelter test; Veloc, mean velocity (cm/s).

Figure 8 shows the PLS-DA in which the descriptive parameters from the shelter, novel tank diving and scototaxis tests are combined for males and females of all strains (first significant component *R*^2^*X* = 0.324, *R*^2^*Y* = 0.154, Q^2^ = 0.136, second non-significant component *R*^2^*X* = 0.102, *R*^2^*Y* = 0.0716, Q^2^ = 0.011). The combined picture is consistent with that seen for individual tests and shows that the wild fish are located to the right and thus display a consistent pattern across all three tests; characterized by immobility and shelter seeking behavior. The close location of wild males and females indicates that there are no marked sex differences. The AB and *fgfr1a*^−/−^ fish are characterized by distance and velocity measures, duration moving and zone transitions. The close location of AB males and females indicates that there are no marked sex differences, while *fgfr1a*^−/−^ males and females are more separated, with *fgfr1a*^−/−^ males characterized by zone transitions and higher activity in the top zone of the novel tank diving test (Figure 8).

## Discussion

The present study aimed at studying behavioral characteristics related to the concept of boldness or shyness in adult male and female domesticated AB fish, genetically modified *fgfr1a*^−/−^ fish and offspring of wild-caught zebrafish using three common behavioral tests, i.e., the shelter, novel tank diving and scototaxis tests. Overall, the results show that offspring of wild-caught zebrafish are less bold than fish of the AB and genetically modified strain. Fish of the domesticated AB strain and the modified *fgfr1a*^−/−^ strain are similar in boldness, even though strain-related differences in boldness vary between tests.

The tests used herein are all based on forced exploration of areas associated with preferred and non-preferred environments. High activity and exploration in the arena and activity in the non-preferred environment, i.e., increased risk taking, are behaviors that usually are considered in the interpretation of boldness (Collier et al., 2017). The shelter test is based on the conflict of exploring an open half vs. a sheltered half of an open field arena, where boldness is related to activity in the open area (Sneddon, 2003; Dahlbom et al., 2011). The novel tank diving test is based on the natural tendency of zebrafish to initially dive to the bottom of a novel experimental tank, with a gradual increase in vertical activity over time (Gerlai et al., 2000; Levin et al., 2007; Egan et al., 2009; Kysil et al., 2017), and bold fish are characterized by high levels of activity and exploration of the arena and high activity in the top zone (Thörnqvist et al., 2019). In the scototaxis test, also known as the light/dark preference test, the fish is faced with the conflict of exploring a white compartment vs. remaining in the preferred black compartment (Serra et al., 1999; Maximino et al., 2010b; Kysil et al., 2017) and high activity in the white compartment is considered to reflect boldness.

A consistent finding in the present study was that offspring of wild-caught fish were less active in the arenas and displayed lower activity in the non-preferred zone of the respective tests relative to AB and *fgfr1a*^−/−^ fish. This pattern was especially evident in the novel tank diving test in which only six out of nine wild males and four out of nine females visited the top zone, and the majority of time was spent in the bottom zone. This finding thus implies that wild fish are shyer than AB and *fgfr1a*^−/−^ fish and is in line with the report of wild-caught zebrafish as “highly anxious” (Kalueff et al., 2014). Moreover, using a recently established multivariate behavior test, we could demonstrate that male AB fish were more active, explorative and risk taking, i.e., bolder, than wild-caught males (Roman et al., 2016). In a previous study the open field, shelter and novel object tests were used to screen for boldness in wild-caught fish, and the results revealed that bolder individuals were more likely to become dominant in a dyadic contest (Dahlbom et al., 2011), showing that the boldness-shyness continuum is apparent also among wild-caught zebrafish but affected by environmental factors, as the boldness trait was more evident in wild-caught fish from high-predation streams (Roy and Bhat, 2018b).

When comparing behavior in the shelter test between male AB and *fgfr1a*^−/−^ fish it was evident that AB males displayed higher activity than *fgfr1a*^−/−^ males in the total arena and made more visits to the open and sheltered zones. However, no difference was found for specific risk-taking behaviors, such as duration, percentage duration and duration per visit in the open area between AB and *fgfr1a*^−/−^ males. Thus, in this test differences in activity, but not behavioral characteristics associated with boldness, were evident. In contrast, *fgfr1a*^−/−^ males displayed higher activity, spent more time and moved a longer distance in the top zone of the novel tank diving test, implying a bolder behavior in this test relative to AB males. In the scototaxis test, AB males spent more time in the white than in the black compartment and a similar trend was observed in females. Similarly, *fgfr1a*^−/−^ females spent more time in the white compartment whereas *fgfr1a*^−/−^ males spent equal time in the two compartments of the scototaxis arena. Boldness of the *fgfr1a*^−/−^ fish has to our knowledge not previously been compared to that of AB or offspring of wild-caught zebrafish. However, when compared with the Tübingen wild-type, *fgfr1a*^−/−^ fish were found to spend more time close to the novel object and were more explorative and spent longer time on the non-preferred side of the biased place preference tank, indicating a bolder behavior in the *fgfr1a*^−/−^ mutants (Norton et al., 2011). Norton et al. (2011) also reported that in mirror tests, *fgfr1a*^−/−^ mutants were more aggressive than Tübingen wild-type fish. Recently, it was shown that *fgfr1a*^−/−^ males were more aggressive than AB males, but only if tested in a mirror test (Mustafa et al., 2019). In dyadic fights, *fgfr1a*^−/−^ males were not more aggressive than AB males, and they had no advantage in fights for social dominance with size-matched AB males (Mustafa et al., 2019). Boldness is often correlated with aggression, forming a behavioral syndrome (Sih et al., 2004). However, even though the results from the novel tank diving test in the current study show that *fgfr1a*^−/−^ mutants are bolder than zebrafish of the AB strain, it is less clear if they are more aggressive, at least when facing a real opponent.

No differences between male and female offspring of wild-caught fish were detected in any of the tests used. Thus, differences between males and females were highly dependent on strain and test. In the novel tank diving test, *fgfr1a*^−/−^ males were more active and had higher activity and spent more time in the top zone than *fgfr1a*^−/−^ females. Thus, here *fgfr1a*^−/−^ males were bolder than the females and only minor sex differences, of little relevance for interpretation of boldness, were detected in AB fish. In contrast, AB males were more active and had higher activity and spent more time in the open area of the shelter test relative to AB females. Thus, here AB males were bolder than the females and only minor sex differences, of little relevance for interpretation of boldness, were detected in *fgfr1a*^−/−^ fish. Sex difference in boldness observed in the scototaxis test is less clear. In this test, AB males spent significantly more time in the white than in the black compartment whereas in *fgfr1a*^−/−^ fish, the opposite pattern with females spending more time in the white compartment was observed. However, AB females showed a similar trend to spend more time in the white compartment as the AB males, and *fgfr1a*^−/−^ males were indifferent spending equal time in the white and black compartment. A recent study using a multivariate test for behavioral profiling revealed that AB males were bolder than AB females (Roman et al., 2016), but considering the findings herein, that result could be test-specific. A recent study showed that wild-caught males were bolder than females, but boldness was also dependent on the level of predation pressure; boldness in males was especially evident in fish from high-predation streams (Roy and Bhat, 2018b). We expected sex differences to be especially evident in offspring of wild-caught fish, in line with the summary by Genario et al. (2019), and fewer differences in AB and *fgfr1a*^−/−^ fish. This was not the case and it may be that when kept in an artificial environment in the lab, differences depending on environmental factors such as tank density and level of enrichment have a different impact on males and females; thereby driving sex differences to be more pronounced in AB and *fgfr1a*^−/−^ fish. A number of different zebrafish strains are available (Maximino et al., 2010a; Kalueff et al., 2014). The AB strain is among the most common lab strains used. Since AB fish often are bred at different facilities, differences between fish from different facilities can be expected to the same extent as those described in rodents from different vendors (Palm et al., 2011a,b, 2012; Goepfrich et al., 2013; Momeni et al., 2015; Åhlgren and Voikar, 2019). Considering the different tests used, fewer differences were revealed using the scototaxis test. This was supported by the loading in the PLS-DA where the groups appeared closer to each other relative to the pattern obtained from the novel tank diving test and especially the shelter test where the lines loaded further away from each other but with males and females together within the respective strains. It has been suggested that adult zebrafish show a robust dark preference in the scototaxis test and that the operational interpretations of anxiety converge on the same zebrafish behavior (Kysil et al., 2017). The results from our study seem to question this suggestion. Moreover, from our results it can be concluded that the choice of test has a large impact on assessment of behaviors related to boldness, something that has been demonstrated in previous studies where the interpretation of boldness is highly contextual and varies not only between individuals but also between tests (Moretz et al., 2007; Burns, 2008; Toms et al., 2010; Ólafsdóttir and Magellan, 2016). This finding is not unique for zebrafish as similar findings have been obtained in rodent studies. For instance, in a study assessing inbred strains of mice on the elevated-plus maze, light/dark transition box (equivalent to the scototaxis test used herein) and open field test strain differences were found on all measures of locomotor activity and activity in the non-preferred areas, i.e., risk taking (O’Leary et al., 2013). Moreover, strain means for measures of locomotor activity on the three apparatus were significantly correlated while strain means for commonly used measures of risk-taking behavior used for interpretation of anxiety were not correlated (O’Leary et al., 2013).

A limitation in the present study is that the fish were not individually tagged and it was therefore not possible to trace individual behavioral characteristics across tests. Moreover, it cannot be excluded that the order of tests and the fact that a test battery was used influenced the results. Even though it has been suggested that zebrafish may be relatively less sensitive to the test battery effect (Song et al., 2016), it is known from the literature on rodents that test batteries and order of tests can have a profound impact on results (McIlwain et al., 2001; Paylor et al., 2006; Blokland et al., 2012).

In conclusion, profound strain differences have been reported in a variety of behavioral studies using zebrafish (Maximino et al., 2013; Kalueff et al., 2014; Volgin et al., 2019). Here we demonstrate that behavioral characteristics including those associated with boldness differ in a complex pattern depending on strain and test. Therefore, a careful assessment of various strains of fish using both males and females is warranted in order to strengthen the interpretation of results. This is especially important in studies where zebrafish are used as model organisms for human conditions as well as studies evaluating the effects of pharmacological or toxicological substances on behavior.

## Data Availability Statement

The raw data supporting the conclusions of this manuscript will be made available by the authors, without undue reservation, to any qualified researcher.

## Ethics Statement

The animal study was reviewed and approved by Uppsala Regional Animal Ethical Committee.

## Author Contributions

AM and SW contributed to conception and design of the study. AM performed the experiments. AM, ER and SW contributed to the statistical analyses and production of the final version of the manuscript. All authors read and approved the submitted version.

## Conflict of Interest

The authors declare that the research was conducted in the absence of any commercial or financial relationships that could be construed as a potential conflict of interest.

## Footnotes

^1^www.scilifelab.se
