# Supplementary material for: Boldness in Male and Female Zebrafish (Danio rerio) Is Dependent on Strain and Test
Source: Front Behav Neurosci. 2019 Nov 19;13:248. doi: 10.3389/fnbeh.2019.00248 (PMC6877474; doi:10.3389/fnbeh.2019.00248)
Supplement: Supplementary file 1 [file Table_1.docx]

Supplementary Material

Boldness in male and female zebrafish (*Danio rerio*) is dependent on strain and test

Arshi Mustafa^1,2^, Erika Roman^3,4,#^, Svante Winberg^1,#,*^

^1^ Department of Neuroscience, Behavioral Neuroendocrinology Group, Uppsala University, P.O. Box 593, SE-751 24 Uppsala, Sweden

^2^ Department of Organismal Biology, Uppsala University, Norbyvägen 18A, SE-752 36, Sweden

^3^ Department of Pharmaceutical Biosciences, Division of Pharmacology, Neuropharmacology, Addiction and Behavior Unit, Uppsala University, P.O. Box 591, SE-751 24 Uppsala, Sweden

^4^ Department of Anatomy, Physiology and Biochemistry, Division of Anatomy and Physiology, Swedish University of Agricultural Sciences, P.O. Box 7011, SE-750 07 Uppsala, Sweden

**^#^ Shared senior authorship**

*** Correspondence:**Corresponding Author
svante.winberg@neuro.uu.se


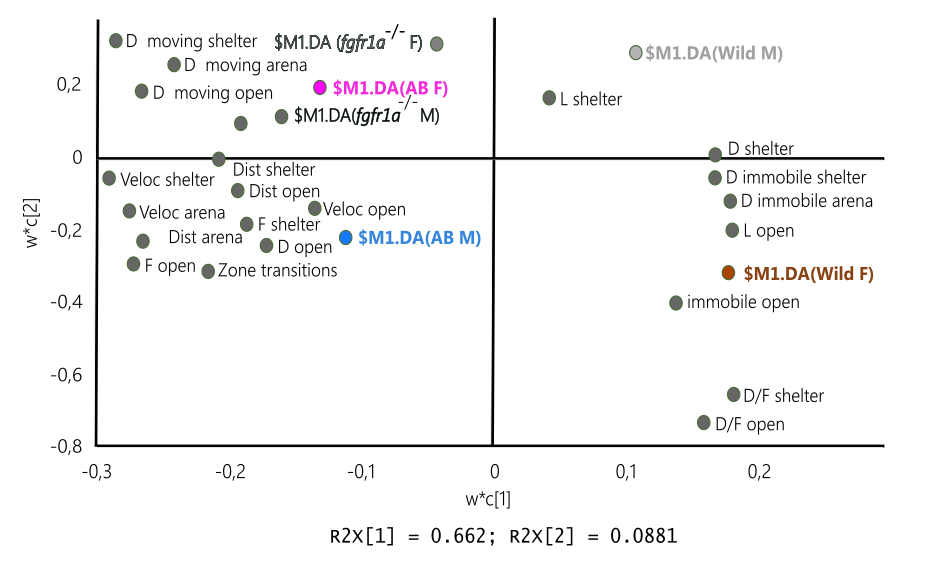


**Supplementary Figure 1**. Scatter plot of variable loadings from the PLS-DA from the shelter test in male (M) and female (F) adult offspring of wild-caught (wild), AB and *fgfr1a ^-/^* zebrafish. The model generated one significant component (R^2^X=0.662, R^2^Y=0.145, Q^2^=0.131) and a second, non-significant component (R^2^X=0.08, R^2^Y=0.0468, Q^2^= -0.022) was added to obtain a two-dimensional model for visualization.

Abbreviations: D, duration (s); D/F, duration per visit (s); Dist, total distance (cm); F, frequency; L, latency (s); Veloc, mean velocity (cm/s).


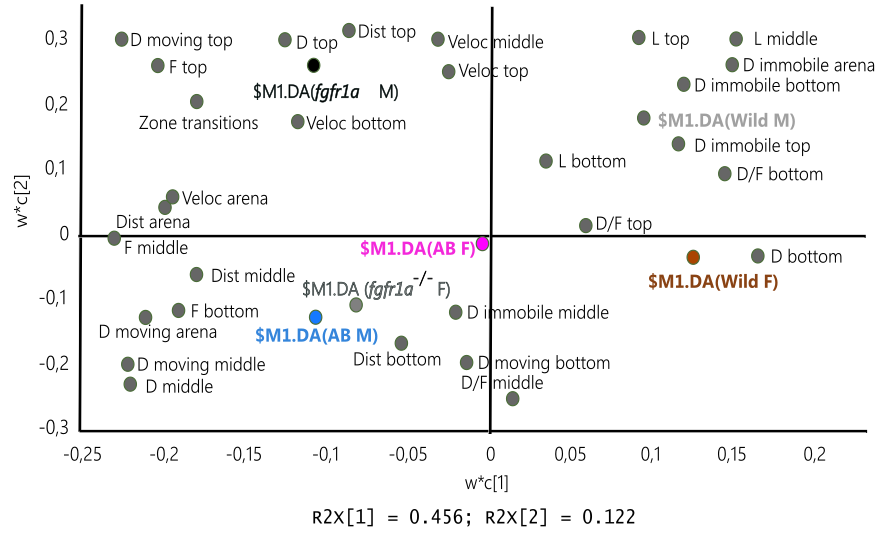


**Supplementary Figure 2.** Scatter plot of variable loadings from the PLS-DA (three significant components, R^2^X(cum)=0.713, R^2^Y=0.213 (cum), Q^2^ (cum)=0.0861) of the novel tank diving test in male (M) and female (F) adult offspring of wild-caught (wild), AB and *fgfr1a ^-/-^* zebrafish.

Abbreviations: D, duration (s); D/F, duration per visit (s); Dist, total distance moved (cm); F, frequency; L, latency (s); Veloc, mean velocity (cm/s).


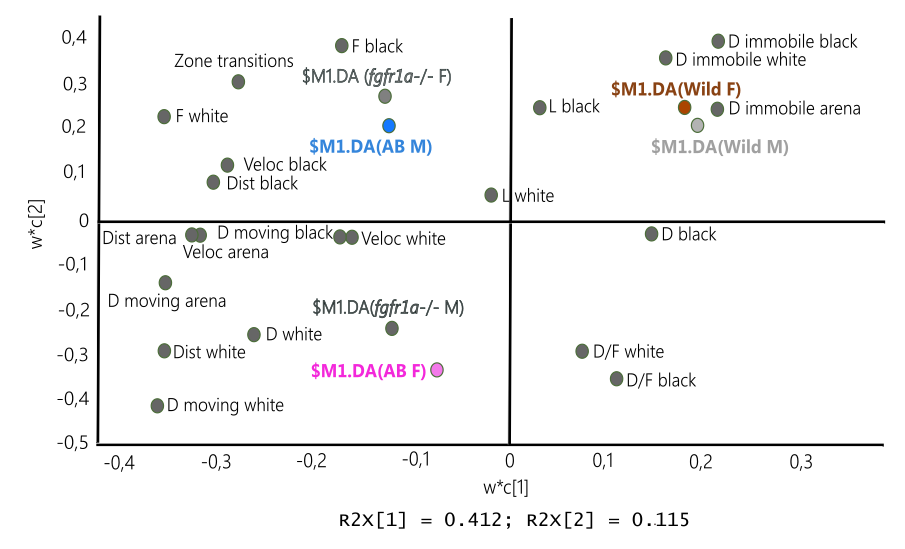


**Supplementary Figure 3**. Scatter plot of variable loadings from the PLS-DA (no significant component; first component (R^2^X=0.412, R^2^Y=0.072, Q^2^=0.035), second component (R^2^X=0.115, R^2^Y = 0.06, Q^2^=-0.003) of the scototaxis test in male (M) and female (F) adult offspring of wild-caught (wild), AB and *fgfr1a ^-/-^* zebrafish.

Abbreviations: D, duration (s); D/F, duration per visit (s); Dist, total distance (cm); F, frequency; L, latency (s); Veloc, mean velocity (cm/s).

# Supplementary Table 1. Behavioral data from the shelter test in male (M) and female (F) adult offspring of wild-caught (wild), AB and *fgfr1a ^-/-^* zebrafish.

| **Zone** | **Sex** | **Wild** | **AB** | ***fgfr1a ^-/-^*** |
| --- | --- | --- | --- | --- |
| **Open area** |  |  |  |  |
| Occurrence | M | 9/9 | 9/9 | 9/9 |
|  | F | 6/9 | 9/9 | 9/9 |
|  |  |  |  |  |
| Latency (s) | M | 104.0 (279.5) | 60.0 (4.0) ww | 60.0 (4.0) ww |
|  | F | 172.5 (577.3) | 67.0 (19.5) | 61.0 (18.5) |
|  |  |  |  |  |
| Frequency | M | 30.0 (36.0) | 78.0 (31.0) *, www | 67.0 (14.5) ww, a |
|  | F | 13.0 (22.8) | 68.0 (25.5) ww | 56.0 (27.5) ww |
|  |  |  |  |  |
| Duration (s) | M | 246.2 (434.9) | 472.6 (206.9) | 391.2 (121.9) |
|  | F | 261.9 (460.2) | 413.6 (197.2) | 434.8 (179.6) |
|  |  |  |  |  |
| Duration per visit (s) | M | 5.7 (7.3) | 5.1 (3.1) | 5.3 (2.1) |
|  | F | 9.9 (24.5) | 6.1 (2.9) | 6.1 (4.2) |
|  |  |  |  |  |
| Distance (cm) | M | 398.0 (1050.1) | 1675.1 (1186.5) www | 1473.4 (468.8) w |
|  | F | 227.4 (547.9) | 1307.8 (555.2) ww | 1432.1 (931.0) ww |
|  |  |  |  |  |
| Velocity (cm/s) | M | 2.5 (1.7) | 4.7 (1.8) *, ww | 3.4 (0.8) |
|  | F | 2.5 (2.0) | 3.3 (1.6) w | 3.0 (0.8) |
|  |  |  |  |  |
| Duration moving (s) | M | 91.8 (251.3) | 346.8 (162.9) ww | 350.4 (107.6) ww |
|  | F | 52.4 (116.2) | 346.8 (158.9) ww | 382.6 (207.6) ww |
|  |  |  |  |  |
| Duration immobile (s) | M | 75.0 (200.3) | 46.0 (96.2) | 50.8 (20.4) |
|  | F | 89.5 (404.3) | 46.4 (75.6) | 59.4 (87.0) |
|  |  |  |  |  |
| **Sheltered area** |  |  |  |  |
| Latency (s) | M | 60.0 (0.0) | 60.0 (2.4) | 61.6 (5.3) |
|  | F | 60.0 (0.0) | 60.0 (3.0) | 60.0 (2.9) |
|  |  |  |  |  |
| Frequency | M | 31.0 (35.0) | 79.0 (29.5) *, www | 67.0 (14.0) ww, a |
|  | F | 7.0 (16.5) | 69.0 (26.0) www | 55.0 (27.5) www |
|  |  |  |  |  |
| Duration (s) | M | 593.8 (434.9) | 367.4 (206.9) | 448.8 (121.9) |
|  | F | 776.2 (445.2) | 426.4 (197.2) | 405.2 (179.6) |
|  |  |  |  |  |
| Duration per visit (s) | M | 21.8 (177.5) | 4.7 (3.9) | 6.2 (3.7) |
|  | F | 110.9 (822.6) | 6.2 (4.4) | 5.8 (5.6) |
|  |  |  |  |  |
| Distance (cm) | M | 752.5 (507.3) * | 1530.8 (1376.8) ww | 1660.3 (480.0) **, ww |
|  | F | 255.4 (371.1) | 1694.9 (928.3) www | 1246.8 (618.2) www |
|  |  |  |  |  |
| Velocity (cm/s) | M | 1.3 (2.1) | 4.7 (1.6) ww | 3.7 (1.0) *, ww |
|  | F | 0.3 (1.4) | 3.4 (1.5) www | 2.9 (1.6) www |
|  |  |  |  |  |
| Duration moving (s) | M | 172.2 (132.5) | 310.6 (228.1) ww | 393.8 (70.0) **, www |
|  | F | 51.4 (103.8) | 389.2 (155.1) www | 307.6 (128.1) www |
|  |  |  |  |  |
| Duration immobile (s) | M | 421.6 (626.6) | 48.2 (41.1) ww | 40.6 (51.5) ww |
|  | F | 724.8 (625.9) | 44.4 (54.4) www | 82.6 (168.7) ww |
|  |  |  |  |  |
| **Activity** |  |  |  |  |
| Total activity | M | 61.0 (71.0) | 157.0 (60.5) *, www | 133.0 (28.5) ww, a |
|  | F | 13.0 (34.0) | 137.0 (51.5) www | 111.0 (55.0) www |
|  |  |  |  |  |
| Distance arena (cm) | M | 1154.8 (1487.0) | 3922.3 (1517.1) www | 3007.2 (735.5) *, ww |
|  | F | 432.9 (734.9) | 2660.4 (1323.7) www | 2259.0 (1007.4) www |
|  |  |  |  |  |
| Velocity arena (cm/s) | M | 1.4 (1.8) | 4.7 (1.8) www | 3.6 (0.9) *, ww |
|  | F | 0.5 (0.9) | 3.2 (1.6) www | 2.7 (1.2) www |
|  |  |  |  |  |
| Duration moving arena (s) | M | 267.8 (371.2) | 739.0 (107.2) www | 733.6 (34.7) www |
|  | F | 95.4 (177.7) | 728.4 (96.8) www | 633.8 (222.0) www |
|  |  |  |  |  |
| Duration immobile arena (s) | M | 572.2 (371.2) | 101.0 (107.1) www | 106.4 (34.7) www |
|  | F | 744.6 (177.7) | 111.6 (96.8) www | 206.2 (221.9) www |
|  |  |  |  |  |
| Zone transitions open to shelter | M | 11.0 (12.5) | 43.0 (27.0) www | 40.0 (10.5) ww |
|  | F | 2.0 (4.5) | 30.0 (13.5) www | 19.0 (26.5) ww |
|  |  |  |  |  |
| Zone transitions shelter to open | M | 11.0 (13.5) * | 43.0 (29.0) www | 41.0 (10.5) ww |
|  | F | 1.0 (5.5) | 29.0 (13.0) www | 19.0 (26.5) ww |

Data are presented as median (quartile range). If a fish did not visit a zone this was considered a missing value in the statistical analysis and occurrence (fish entering a zone/number of tested fish) is shown for the zones that were not visited by all fish. ^w^p<0.05, ^ww^p<0.01, ^www^p<0.001 compared to wild zebrafish within the respective sex; ^a^p<0.05 compared to AB fish within the respective sex; *p<0.05, **p<0.01 compared to females within the respective line (Mann-Whitney U-test).

**Supplementary Table 2.** Summary of statistical analyses of behavioral data from the shelter test in male (M) and female (F) adult offspring of wild-caught (wild), AB and *fgfr1a ^-/-^* zebrafish.

| **Open area** |  | **Wild vs AB** | **Wild vs *fgfr1a ^-/-^*** | **AB vs *fgfr1a ^-/-^*** | **KW** |
| --- | --- | --- | --- | --- | --- |
| Latency (s) | M | U=6.5; p<0.01 | U=11.5; p<0.01 | n.s. | H=11.3, N=27; p<0.01 |
|  | F | n.s. | n.s. | n.s. | n.s. |
|  |  |  |  |  |  |
| Occurrence | M | 9/9 | 9/9 | 9/9 |  |
|  | F | 6/9 | 9/9 | 9/9 |  |
|  |  |  |  |  |  |
| Frequency | M | U=2.0; p≤0.001 | U=7.0; p<0.01 | U=16.0; p<0.05 | H=16.3, N=27; p<0.001 |
|  | F | U=1.5; p<0.01 | U=2.5; p<0.01 | n.s. | H=11.5, N=24; p<0.010 |
|  |  |  |  |  |  |
| Duration (s) | M | n.s. | n.s. | n.s. | n.s. |
|  | F | n.s. | n.s. | n.s. | n.s. |
|  |  |  |  |  |  |
| Duration per visit (s) | M | n.s. | n.s. | n.s. | n.s. |
|  | F | n.s. | n.s. | n.s. | n.s. |
|  |  |  |  |  |  |
| Distance (cm) | M | U=4.0; p≤0.001 | U=12.00; p<0.05 | n.s. | H=12.5, N=27; p<0.01 |
|  | F | U=2.0; p<0.01 | U=4.0, p<0.01 | n.s. | H=10.3, N=24; p<0.01 |
|  |  |  |  |  |  |
| Velocity (cm/s) | M | U=9.0; p<0.01 | n.s. | n.s. | H=9.0, N= 27; p<0.05 |
|  | F | U=10.0; p<0.05 | n.s. | n.s. | n.s. |
|  |  |  |  |  |  |
| Duration moving (s) | M | U=5.0; p<0.01 | U=6.0; p<0.01 | n.s. | H=13.1, N=27; p<0.01 |
|  | F | U=3.0; p<0.01 | U=4.0; p<0.01 | n.s. | H=9.8, N=24; p<0.01 |
|  |  |  |  |  |  |
| Duration immobile (s) | M | n.s. | n.s. | n.s. | n.s. |
|  | F | n.s. | n.s. | n.s. | n.s. |
|  |  |  |  |  |  |
| **Sheltered area** |  |  |  |  |  |
| Latency (s) | M | n.s. | n.s. | n.s. | n.s. |
|  | F | n.s. | n.s. | n.s. | n.s. |
|  |  |  |  |  |  |
| Frequency | M | U=2.0; p≤0.001 | U=7.0; p<0.01 | U=15.5; p<0.05 | H=16.4, N=27; p<0.001 |
|  | F | U=2.0; p≤0.001 | U=2.5; p≤0.001 | n.s. | H=15.8, N=27; p<0.001 |
|  |  |  |  |  |  |
| Duration (s) | M | n.s. | n.s. | n.s. | n.s. |
|  | F | n.s. | n.s. | n.s. | n.s. |
|  |  |  |  |  |  |
| Duration per visit (s) | M | U=5.0; p<0.01 | U=10.5; p<0.01 | n.s. | H=12.8, N=27; p<0.01 |
|  | F | U=2.0; p≤0.001 | U=5.0; p<0.01 | n.s. | H=14.6, N= 27; p<0.001 |
|  |  |  |  |  |  |
| Distance (cm) | M | U=8.0; p<0.01 | U=8.0; p<0.01 | n.s. | H=11.2, N=27; p<0.01 |
|  | F | U=1.0; p <0.001 | U=2.0; p≤0.001 | n.s. | H=17.7, N=27; p<0.001 |
|  |  |  |  |  |  |
| Velocity (cm/s) | M | U=5.0; p<0.01 | U=10.0; p<0.01 | n.s. | H=13.4, N=27; p<0.01 |
|  | F | U=0.0; p<0.001 | U=4.0; p≤0.001 | n.s. | H=17.5, N=27; p<0.001 |
|  |  |  |  |  |  |
| Duration moving (s) | M | U=7.0; p<0.01 | U=3.0; p<0.001 | n.s. | H=13.9, N=27; p<0.001 |
|  | F | U=2.0; p≤0.001 | U=4.0; p≤0.001 | n.s. | H=16.2, N= 27; p<0.001 |
|  |  |  |  |  |  |
| Duration immobile (s) | M | U=5.0; p<0.01 | U=5.0; p<0.01 | n.s. | H=13.4, N=27; p<0.01 |
|  | F | U=3.0; p≤0.001 | U=9.0; p<0.01 | n.s. | H=13.4, N=27; p<0.01 |
|  |  |  |  |  |  |
| **Activity** |  |  |  |  |  |
| Total activity | M | U=2.0; p≤0.001 | U=7.0; p<0.01 | U=16.0; p<0.05 | H=16.3, N=27; p<0.001 |
|  | F | U=3.0; p≤0.001 | U=2.5; p≤0.001 | n.s. | H=15.8, N=27; p<0.001 |
|  |  |  |  |  |  |
| Distance arena (cm) | M | U=3.0; p≤0.001 | U=9.0; p<0.01 | n.s. | H=14.4, N=27; p<0.001 |
|  | F | U=1.0; p<0.001 | U=2.0; p≤0.001 | n.s. | H=17.1, N=27; p<0.001 |
|  |  |  |  |  |  |
| Velocity arena (cm/s) | M | U=3.0; p≤0.001 | U=9.0; p<0.01 | n.s. | H=14.4, N=27; p<0.001 |
|  | F | U=1.0; p<0.001 | U=2.0; p≤0.001 | n.s. | H=17.1, N=27; p<0.001 |
|  |  |  |  |  |  |
| Duration moving arena (s) | M | U=3.0; p≤0.001 | U=1.0; p<0.001 | n.s. | H=15.7, N=27; p<0.001 |
|  | F | U=0.0; p<0.001 | U=4.0; p≤0.001 | n.s. | H=16.7, N=27; p<0.001 |
|  |  |  |  |  |  |
| Duration immobile arena (s) | M | U=3.0; p≤0.001 | U=1.0; p<0.001 | n.s. | H=15.7, N=27; p<0.001 |
|  | F | U=0.0; p<0.001 | U=4.0; p≤0.001 | n.s. | H=16.7, N= 27; p<0.001 |
|  |  |  |  |  |  |
| Zone transitions open to shelter | M | U=4.0; p≤0.001 | U=7.0; p<0.01 | n.s. | H=13.3, N=27; p<0.01 |
|  | F | U=2.0; p≤0.001 | U=5.0; p<0.01 | n.s. | H=15.2, N=27; p<0.001 |
|  |  |  |  |  |  |
| Zone transitions shelter to open | M | U=4.5; p≤0.001 | U=7.5; p<0.01 | n.s. | H=12.9, N=27; p<0.01 |
|  | F | U=2.0; p≤0.001 | U=5.0; p<0.01 | n.s. | H=15.1, N=27; p<0.001 |

# Data from the three different strains were compared within sex using a Kruskal-Wallis analysis of variance (KW) whereas the Mann-Whitney U-test was applied for pairwise comparisons between strains within sex. Values presented are Mann-Whitney U-values (U) and p-values, and Kruskal-Wallis (KW) H-value (H), total number of fish (N) and p-values. Non-significant results are indicated by n.s. If a fish did not visit a zone this was considered a missing value in the statistical analysis and occurrence (fish entering a zone/number of tested fish) is shown for the zones that were not visited by all fish.

**Supplementary Table 3.** Behavioral data from the novel tank diving test in male (M) and female (F) adult offspring of wild-caught (wild), AB and *fgfr1a ^-/-^* zebrafish.

| **Zone** | **Sex** | **Wild** | **AB** | ***fgfr1a ^-/-^*** |
| --- | --- | --- | --- | --- |
| **Bottom zone** |  |  |  |  |
| Frequency | M | 8.0 (28.5) | 26.0 (20.0) *, w | 32.0 (18.5) w |
|  | F | 1.0 (14.5) | 16.0 (9.5) | 34.0 (27.5) w |
|  |  |  |  |  |
| Duration (s) | M | 319.0 (156.0) | 107.0 (96.5) ww | 113.0 (46.5) **, ww |
|  | F | 360.0 (133.0) | 137.0 (248.0) | 215.0 (87.0) |
|  |  |  |  |  |
| Duration per visit (s) | M | 17.7 (353.7) | 4.8 (3.5) *, w | 3.6 (2.6) *, ww |
|  | F | 360.0 (342.5) | 8.3 (26.4) w | 5.8 (14.8) w |
|  |  |  |  |  |
| Distance (cm) | M | 393.0 (1432.5) | 676.0 (519.0) | 673.0 (541.0) * |
|  | F | 707.0 (990.5) | 685.0 (673.5) | 1535.0 (909.5) w, a |
|  |  |  |  |  |
| Velocity (cm/s) | M | 4.0 (7.0) | 6.0 (3.5) | 7.0 (2.5) |
|  | F | 3.0 (3.5) | 5.0 (2.5) w | 6.0 (2.0) ww |
|  |  |  |  |  |
| Duration moving (s) | M | 76.0 (200.5) | 107.0 (89.5) | 108.0 (41.5) ** |
|  | F | 125.0 (195.5) | 130.0 (111.0) | 204.0 (96.0) |
|  |  |  |  |  |
| Duration immobile (s) | M | 111.0 (350.0) | 4.0 (8.0) ww | 6.0 (6.0) **, w |
|  | F | 190.0 (233.0) | 9.0 (108.5) w | 16.0 (9.5) www |
|  |  |  |  |  |
| **Middle zone** |  |  |  |  |
| Occurrence | M | 6/9 | 9/9 | 9/9 |
|  | F | 4/9 | 8/9 | 8/9 |
|  |  |  |  |  |
| Latency | M | 106.8 (169.4) | 19.6 (17.8) ww | 27.0 (33.3) w |
|  | F | 140.9 (204.0) | 24.6 (44.3) | 28.8 (31.1) |
|  |  |  |  |  |
| Frequency | M | 32.0 (41.5) | 55.0 (43.5) w | 81.0 (43.5) ww, a |
|  | F | 26.5 (61.5) | 41.1 (50.5) | 59.5 (33.8) |
|  |  |  |  |  |
| Duration (s) | M | 47.5 (81.8) | 136.0 (76.5) ww | 101.0 (33.5) w, a |
|  | F | 47.0 (83.3) | 108.5 (98.5) | 96.0 (29.0) |
|  |  |  |  |  |
| Duration per visit (s) | M | 1.4 (0.8) | 2.2 (1.2) w | 1.2 (0.4) *, aa |
|  | F | 1.6 (0.9) | 1.5 (2.1) | 1.5 (0.5) |
|  |  |  |  |  |
| Distance (cm) | M | 377.0 (687.0) | 653.0 (869.0) | 868.0 (479.0) |
|  | F | 240.5 (506.8) | 545.5 (899.5) | 749.5 (477.0) |
|  |  |  |  |  |
| Velocity (cm/s) | M | 9.5 (9.0) | 6.0 (4.5) | 8.0 (2.5) |
|  | F | 5.5 (3.3) | 9.5 (6.3) | 8.5 (3.8) |
|  |  |  |  |  |
| Duration moving (s) | M | 47.0 (74.5) | 131.0 (89.5) w | 99.0 (33.0) w |
|  | F | 42.5 (73.3) | 101.0 (96.0) | 92.5 (29.5) |
|  |  |  |  |  |
| Duration immobile (s) | M | 0.5 (3.3) | 2.0 (15.0) | 2.0 (1.5) |
|  | F | 4.5 (9.3) | 0.5 (6.8) | 1.0 (1.8) |
|  |  |  |  |  |
| **Top** |  |  |  |  |
| Occurrence | M | 6/9 | 9/9 | 9/9 |
|  | F | 4/9 | 8/9 | 8/9 |
|  |  |  |  |  |
| Latency | M | 85.7 (186.9) | 24.8 (15.3) | 65.2 (77.3) |
|  | F | 154.0 (142.6) | 35.6 (147.8) | 41.1 (51.1) |
|  |  |  |  |  |
| Frequency | M | 15.5 (17.3) | 27.0 (26.5) | 60.0 (27.0) **, ww, aa |
|  | F | 17.0 (38.3) | 25.0 (41.5) | 21.0 (14.0) |
|  |  |  |  |  |
| Duration (s) | M | 34.0 (136.5) | 105.0 (46.5) | 139.0 (66.0) **, w, a |
|  | F | 86.0 (69.8) | 112.5 (121.5) | 33.0 (40.0) |
|  |  |  |  |  |
| Duration per visit (s) | M | 1.4 (6.7) | 2.8 (1.9) | 3.0 (1.6) |
|  | F | 2.8 (5.8) | 2.8 (3.9) | 1.9 (1.8) |
|  |  |  |  |  |
| Distance (cm) | M | 271.5 (1529.5) | 545.0 (451.0) | 1102.0 (440.0) **, w, aa |
|  | F | 273.0 (482.3) | 543.0 (993.0) | 266.0 (202.3) |
|  |  |  |  |  |
| Velocity (cm/s) | M | 11.5 (5.5) * | 6.0 (4.5) | 8.0 (4.0) |
|  | F | 4.0 (3.5) | 9.0 (9.5) | 8.5 (3.8) |
|  |  |  |  |  |
| Duration moving (s) | M | 34.0 (73.8) | 80.0 (38.0) | 137.0 (37.0) **, ww, aa |
|  | F | 39.0 (68.5) | 110.5 (107.3) | 32.5 (39.3) |
|  |  |  |  |  |
| Duration immobile (s) | M | 0.5 (63.5) | 2.0 (20.0) | 4.0 (3.0) *** |
|  | F | 21.5 (53.5) | 2.5 (6.0) | 0.5 (1.0) |
|  |  |  |  |  |
| **Activity** |  |  |  |  |
| Total activity | M | 41.0 (88.5) | 110.0 (86.0) ww | 170.0 (89.5) *, www, a |
|  | F | 1.0 (57.0) | 79.0 (111.5) | 111.0 (102.0) w |
|  |  |  |  |  |
| Distance arena (cm) | M | 1720.0 (3112.5) | 2310.0 (1436.5) | 2605.0 (947.0) |
|  | F | 1044.0 (1280.0) | 1736.0 (1615.0) w | 2817.0 (1009.0) www |
|  |  |  |  |  |
| Velocity arena (cm/s) | M | 5.0 (8.5) | 6.0 (3.5) | 7.0 (3.0) |
|  | F | 3.0 (3.5) | 5.0 (4.5) w | 8.0 (3.0) www |
|  |  |  |  |  |
| Duration moving arena (s) | M | 125.0 (325.5) | 350.0 (41.0) w | 349.0 (8.5) ww |
|  | F | 169.0 (257.0) | 325.0 (106.5) w | 344.0 (12.5) www |
|  |  |  |  |  |
| Duration immobile arena (s) | M | 235.0 (325.5) | 10.0 (41.0) w | 11.0 (8.5) ww |
|  | F | 191.0 (257.0) | 35.0 (106.5) w | 16. 0 (12.5) www |
|  |  |  |  |  |
| Zone transitions bottom-middle-top | M | 1.0 (4.0) | 4.0 (7.0) | 6.0 (4.0) |
|  | F | 0.0 (4.0) | 3.0 (3.5) | 4.0 (4.5) |
|  |  |  |  |  |
| Zone transitions top-middle-bottom | M | 0.0 (4.5) | 3.0 (4.5) w | 11.0 (9.0) ww, aa |
|  | F | 0.0 (4.5) | 6.0 (8.0) | 7.0 (7.5) |
|  |  |  |  |  |
| Zone transitions bottom-middle | M | 2.0 (11.5) | 9.0 (4.5) | 19.0 (10.5) ww, aaa |
|  | F | 0.0 (9.0) | 9.0 (10.0) | 12.0 (12.5) |
|  |  |  |  |  |
| Zone transitions middle-bottom | M | 2.0 (11.5) | 9.0 (5.0) | 18.0 (10.5) ww, aa |
|  | F | 0.0 (8.0) | 9.0 (10.5) | 12.0 (13.0) |
|  |  |  |  |  |
| Zone transitions bottom-top | M | 7.0 (12.0) | 15.0 (7.5) w | 24.0 (8.5) www, a |
|  | F | 0.0 (12.5) | 11.0 (11.5) | 15.0 (15.0) |
|  |  |  |  |  |
| Zone transitions top-bottom | M | 6.0 (11.5) | 15.0 (6.5) w | 23.0 (8.5) www, a |
|  | F | 0.0 (12.0) | 10.0 (11.5) | 14.0 (15.5) |
|  |  |  |  |  |
| Zone transitions middle-top | M | 3.0 (10.0) | 9.0 (5.0) | 23.0 (12.5) *, www, aa |
|  | F | 0.0 (9.0) | 12.0 (14.0) | 12.0 (8.5) |
|  |  |  |  |  |
| Zone transitions top-middle | M | 4.0 (9.5) | 9.0 (5.0) | 22.0 (12.5) *, www, aa |
|  | F | 0.0 (8.5) | 12.0 (13.5) | 12.0 (8.0) |

Data are presented as median (quartile range). If a fish did not visit a zone this was considered a missing value in the statistical analysis and occurrence (fish entering a zone/number of tested fish) is shown for the zones that were not visited by all fish. ^w^p<0.05, ^ww^p<0.01, ^www^p<0.001 compared to wild zebrafish within the respective sex; ^a^p<0.05, ^aa^p<0.01 compared to AB fish within the respective sex; *p<0.05, **p<0.01, ***p<0.001 compared to females within the respective line (Mann-Whitney U-test).

**Supplementary Table 4.** Summary of statistical analyses of behavioral data from the novel tank diving test in male (M) and female (F) adult offspring of wild-caught (wild), AB and *fgfr1a ^-/-^* zebrafish.

| **Zone** | **Sex** | **Wild vs AB** | **Wild vs *fgfr1a ^-/-^*** | **AB vs *fgfr1a ^-/-^*** | **KW** |
| --- | --- | --- | --- | --- | --- |
| **Bottom zone** |  |  |  |  |  |
| Frequency | M | U=17.0; p<0.05 | U=13.5; p<0.05 | n.s. | H=6.8, N=27; p<0.05 |
|  | F | n.s. | U=14.5; p<0.05 | n.s. | H=7.3, N=27; p<0.05 |
|  |  |  |  |  |  |
| Duration (s) | M | U=11.0; p<0.01 | U=9.0; p<0.01 | n.s. | H=9.9, N=27; p<0.01 |
|  | F | n.s. | n.s. | n.s. | n.s. |
|  |  |  |  |  |  |
| Duration per visit (s) | M | U=12.0; p<0.05 | U=9.0; p<0.01 | n.s. | H=9.8, N=27; p<0.01 |
|  | F | U=17.5; p<0.05 | U=15.5; p<0.05 | n.s. | H=6.4, N=27; p<0.05 |
|  |  |  |  |  |  |
| Distance (cm) | M | n.s. | n.s. | n.s. | n.s. |
|  | F | n.s. | U=15.0; p<0.05 | U=17.0; p<0.05 | H=6.4, N=27; p<0.05 |
|  |  |  |  |  |  |
| Velocity (cm/s) | M | n.s. | n.s. | n.s. | n.s. |
|  | F | U=15.5; p<0.05 | U=4.5; p≤0.001 | n.s. | H=12.5, N=27; p<0.01 |
|  |  |  |  |  |  |
| Duration moving (s) | M | n.s. | n.s. | n.s. | n.s. |
|  | F | n.s. | n.s. | n.s. | n.s. |
|  |  |  |  |  |  |
| Duration immobile (s) | M | U=10.5; p<0.01 | U=13.5; p<0.05 | n.s. | H=9.3, N=27; p<0.01 |
|  | F | U=13.5; p<0.05 | U=2.5; p≤0.001 | n.s. | H=11.3, N=27; p<0.01 |
|  |  |  |  |  |  |
| **Middle zone** |  |  |  |  |  |
| Occurrence | M | 6/9 | 9/9 | 9/9 |  |
|  | F | 4/9 | 8/9 | 8/9 |  |
|  |  |  |  |  |  |
| Latency | M | U=5.0; p<0.01 | U=10; p<0.05 | n.s. | H=7.5, N=24; p<0.01 |
|  | F | n.s. | n.s. | n.s. | n.s. |
|  |  |  |  |  |  |
| Frequency | M | U=9.0; p<0.05 | U=2.0; p<0.01 | U=17.0; p<0.05 | H=11.5, N=24; p<0.01 |
|  | F | n.s. | n.s. | n.s. | n.s. |
|  |  |  |  |  |  |
| Duration (s) | M | U=4.0; p<0.01 | U=7.0; p<0.05 | U=18.0; p<0.05 | H=10.8, N=24; p<0.01 |
|  | F | n.s. | n.s. | n.s. | n.s. |
|  |  |  |  |  |  |
| Duration per visit (s) | M | U=7.0; p<0.05 | n.s. | U=5.0; p<0.01 | H=11.5, N=24; p<0.01 |
|  | F | n.s. | n.s. | n.s. | n.s. |
|  |  |  |  |  |  |
| Distance (cm) | M | n.s. | n.s. | n.s. | n.s. |
|  | F | n.s. | n.s. | n.s. | n.s. |
|  |  |  |  |  |  |
| Velocity (cm/s) | M | n.s. | n.s. | n.s. | n.s. |
|  | F | n.s. | n.s. | n.s. | n.s. |
|  |  |  |  |  |  |
| Duration moving (s) | M | U=6.0; p<0.05 | U=7.0; p<0.05 | n.s. | H=8.3, N=24; p<0.05 |
|  | F | n.s. | n.s. | n.s. | n.s. |
|  |  |  |  |  |  |
| Duration immobile (s) | M | n.s. | n.s. | n.s. | n.s. |
|  | F | n.s. | n.s. | n.s. | n.s. |
|  |  |  |  |  |  |
| **Top** |  |  |  |  |  |
| Occurrence | M | 6/9 | 9/9 | 9/9 |  |
|  | F | 4/9 | 8/9 | 8/9 |  |
|  |  |  |  |  |  |
| Latency | M | n.s. | n.s. | n.s. | n.s. |
|  | F | n.s. | n.s. | n.s. | n.s. |
|  |  |  |  |  |  |
| Frequency | M | n.s. | U=0.5; p<0.01 | U=9.5; p<0.01 | H=13.8, N=24; p<0.001 |
|  | F | n.s. | n.s. | n.s. | n.s. |
|  |  |  |  |  |  |
| Duration (s) | M | n.s. | U=9.0; p<0.05 | U=14.0; p<0.05 | H=8.3, N=24; p<0.05 |
|  | F | n.s. | n.s. | n.s. | n.s. |
|  |  |  |  |  |  |
| Duration per visit (s) | M | n.s. | n.s. | n.s. | n.s. |
|  | F | n.s. | n.s. | n.s. | n.s. |
|  |  |  |  |  |  |
| Distance (cm) | M | n.s. | U=9.0; p<0.05 | U=8.0; p<0.01 | H=9.3, N=24; p<0.01 |
|  | F | n.s. | n.s. | n.s. | n.s. |
|  |  |  |  |  |  |
| Velocity (cm/s) | M | n.s. | n.s. | n.s. | n.s. |
|  | F | n.s. | n.s. | n.s. | n.s. |
|  |  |  |  |  |  |
| Duration moving (s) | M | n.s. | U=2.0; p<0.01 | U=6.0; p<0.01 | H=14.2, N=24; p<0.001 |
|  | F | n.s. | n.s. | n.s. | n.s. |
|  |  |  |  |  |  |
| Duration immobile (s) | M | n.s. | n.s. | n.s. | n.s. |
|  | F | n.s. | n.s. | n.s. | n.s. |
|  |  |  |  |  |  |
| **Activity** |  |  |  |  |  |
| Total activity | M | U=10.0; p<0.01 | U=2.0; p≤0.001 | U=17.0; p<0.05 | H=15.3, N=27; p<0.001 |
|  | F | n.s. | U=15.5; p<0.05 | n.s. | H=6.4, N=27; p<0.05 |
|  |  |  |  |  |  |
| Distance arena (cm) | M | n.s. | n.s. | n.s. | n.s. |
|  | F | U=16.0; p<0.05 | U=3.0; p≤0.001 | n.s. | H=11.6, N=27; p<0.01 |
|  |  |  |  |  |  |
| Velocity arena (cm/s) | M | n.s. | n.s. | n.s. | n.s. |
|  | F | U=13.5; p<0.05 | U=3.5; p≤0.001 | n.s. | H=12.0, N=27; p<0.01 |
|  |  |  |  |  |  |
| Duration moving arena (s) | M | U=12.0; p<0.05 | U=11.0; p<0.01 | n.s. | H=9.0, N=27; p<0.05 |
|  | F | U=12.0; p<0.05 | U=1.0; p<0.001 | n.s. | H=13.2, N=27; p<0.01 |
|  |  |  |  |  |  |
| Duration immobile arena (s) | M | U=12.0; p<0.05 | U=11.0; p<0.01 | n.s. | H=9.0, N=27; p<0.05 |
|  | F | U=12.0; p<0.05 | U=1.0; p<0.001 | n.s. | H=13.2, N=27; p<0.01 |
|  |  |  |  |  |  |
| Zone transitions bottom-middle-top | M | n.s. | n.s. | n.s. | n.s. |
|  | F | n.s. | n.s. | n.s. | n.s. |
|  |  |  |  |  |  |
| Zone transitions top-middle-bottom | M | U=18.5; p<0.05 | U=6.5; p<0.01 | U=10.5; p<0.01 | H=13.1, N=27; p<0.01 |
|  | F | n.s. | n.s. | n.s. | n.s. |
|  |  |  |  |  |  |
| Zone transitions bottom-middle | M | n.s. | U=5.0; p<0.01 | U=4.0; p≤0.001 | H=14.7, N=27; p<0.001 |
|  | F | n.s. | n.s. | n.s. | n.s. |
|  |  |  |  |  |  |
| Zone transitions middle-bottom | M | n.s. | U=6.0; p<0.01 | U=6.0; p<0.01 | H=13.7, N=27; p<0.001 |
|  | F | n.s. | n.s. | n.s. | n.s. |
|  |  |  |  |  |  |
| Zone transitions bottom-top | M | U=15.5; p<0.05 | U=2.0; p≤0.001 | U=13.0; p<0.05 | H=14.9, N=27; p<0.001 |
|  | F | n.s. | n.s. | n.s. | n.s. |
|  |  |  |  |  |  |
| Zone transitions top-bottom | M | U=3.0; p<0.05 | U=2.5; p≤0.001 | U=12.5; p<0.05 | H=15.3, N=27; p<0.001 |
|  | F | n.s. | n.s. | n.s. | n.s. |
|  |  |  |  |  |  |
| Zone transitions middle-top | M | n.s. | U=4.0; p≤0.001 | U=7.0; p<0.01 | H=14.7, N=27; p<0.001 |
|  | F | n.s. | n.s. | n.s. | n.s. |
|  |  |  |  |  |  |
| Zone transitions top-middle | M | n.s. | U=4.0; p≤0.001 | U=7.0; p<0.01 | H=14.7, N=27; p<0.001 |
|  | F | n.s. | n.s. | n.s. | n.s. |

Data from the three different strains were compared within sex using a Kruskal-Wallis analysis of variance (KW) whereas the Mann-Whitney U-test was applied for pairwise comparisons between strains within sex. Values presented are Mann-Whitney U-values (U) and p-values, and Kruskal-Wallis (KW) H-value (H), total number of fish (N) and p-values. Non-significant results are indicated by n.s. If a fish did not visit a zone this was considered a missing value in the statistical analysis and occurrence (fish entering a zone/number of tested fish) is shown for the zones that were not visited by all fish.

**Supplementary Table 5.** Behavioral data from the scototaxis test in male (M) and female (F) adult offspring of wild-caught (wild), AB and *fgfr1a ^-/-^* zebrafish.

| **Zone** | **Sex** | **Wild** | **AB** | ***fgfr1a*** *^-/-^* |
| --- | --- | --- | --- | --- |
| **White compartment** |  |  |  |  |
| Occurrence | M | 8/9 | 8/8 | 7/9 |
|  | F | 8/9 | 8/9 | 9/9 |
|  |  |  |  |  |
| Latency (s) | M | 137.0 (116.0) | 208.5 (279.8) | 220.0 (365.0) |
|  | F | 183.5 (241.5) | 114.5 (127.0) | 91.0 (176.0) |
|  |  |  |  |  |
| Frequency | M | 12.5 (19.3) | 25.0 (34.8) | 33.0 (31.0) w |
|  | F | 12.5 (14.3) | 20.5 (14.3) | 20.0 (49.0) |
|  |  |  |  |  |
| Duration (s) | M | 275.2 (677.0) | 511.4 (333.6) | 578.2 (527.2) |
|  | F | 487.9 (411.9) | 650.4 (249.7) | 448.6 (263.5) |
|  |  |  |  |  |
| Duration per visit (s) | M | 27.6 (85.5) | 20.7 (29.5) | 13.8 (14.3) |
|  | F | 33.5 (46.5) | 32.5 (54.0) | 10.4 (128.8) |
|  |  |  |  |  |
| Distance (cm) | M | 573.5 (1902.5) | 2433.5 (3481.0) w | 4469.0 (4504.0) w |
|  | F | 1673.0 (2042.0) | 3731.0 (1023.0) w | 2660.0 (2726.0) *, aa |
|  |  |  |  |  |
| Velocity (cm/s) | M | 2.5 (4.8) | 6.0 (2.8) | 8.0 (1.0) w |
|  | F | 6.0 (5.5) | 6.0 (1.0) | 6.0 (4.4) |
|  |  |  |  |  |
| Duration moving (s) | M | 52.0 (276.0) | 413.0 (221.5) w | 459.0 (434.5) w |
|  | F | 191.0 (259.3) | 570.0 (190.0) w | 414.0 (395.5) aa |
|  |  |  |  |  |
| Duration immobile (s) | M | 196.2 (648.6) | 36.8 (69.5) | 8.0 (32) w |
|  | F | 120.7 (561.0) | 17.5 (108.7) | 21.0 (45.5) |
|  |  |  |  |  |
| **Black compartment** |  |  |  |  |
| Occurrence | M | 8/9 | 8/8 | 9/9 |
|  | F | 9/9 | 8/9 | 9/9 |
|  |  |  |  |  |
| Latency (s) | M | 60.0 (37.5) | 60.0 (9.3) | 60.0 (0.0) |
|  | F | 60.0 (0.0) | 60.0 (3.8) | 60.0 (31.0) |
|  |  |  |  |  |
| Frequency | M | 12.5 (19.8) | 25.0 (35.3) | 33.0 (35.0) |
|  | F | 12.0 (13.5) | 20.0 (15.0) | 20.0 (50.0) |
|  |  |  |  |  |
| Duration (s) | M | 700.3 (452.8) | 328.6 (333.6) | 407.4 (623.6) |
|  | F | 365.0 (556.0) | 246.4 (577.6) | 391.4 (263.5) |
|  |  |  |  |  |
| Duration per visit (s) | M | 53.3 (258.7) | 7.3 (21.9) | 12.4 (456.7) |
|  | F | 15.4 (98.6) | 10.1 (18.7) | 7.7 (36.2) |
|  |  |  |  |  |
| Distance (cm) | M | 584.5 (3019.3) | 1847.0 (2614.5) | 2069.0 (2553.5) |
|  | F | 1004.0 (1491.0) | 1417.0 (3156.5) | 3021.0 (2499.0) |
|  |  |  |  |  |
| Velocity (cm/s) | M | 4.5 (13.0) | 9.0 (7.0) | 7.0 (4.5) |
|  | F | 3.0 (3.2) | 5.5 (2.8) w | 8.0 (3) w |
|  |  |  |  |  |
| Duration moving (s) | M | 61.5 (461.0) | 282.0 (310.8) | 327.0 (490) |
|  | F | 98.0 (234.0) | 184.5 (592.0) | 387.0 (402) |
|  |  |  |  |  |
| Duration immobile (s) | M | 160.6 (519.9) | 26.5 (81.3) | 11.0 (152.5) |
|  | F | 129.0 (487.0) | 37.0 (78.5) w | 16.3 (57.1) w |
|  |  |  |  |  |
| **Activity** |  |  |  |  |
| Total activity | M | 14.0 (41.0) | 50.0 (70.0) | 65.0 (70.5) |
|  | F | 24.0 (27.5) | 38.0 (42.0) | 40.0 (99.0) |
|  |  |  |  |  |
| Distance arena (cm) | M | 1074.4 (4442.9) | 4896.9 (3768.0) | 6240.4 (2916.5) w |
|  | F | 1901.6 (3654.6) | 4769.1 (1376.1) | 4704.4 (2057.7) |
|  |  |  |  |  |
| Velocity arena (cm/s) | M | 1.3 (5.3) | 5.8 (4.5) | 7.4 (3.5) w |
|  | F | 2.3 (4.4) | 5.7 (1.6) | 5.6 (2.5) |
|  |  |  |  |  |
| Duration moving arena (s) | M | 135.0 (576.5) | 734.0 (236.8) w | 798.0 (264.5) ww |
|  | F | 156.0 (548.5) | 766.0 (147.5) ww | 798.0 (164.0) ww |
|  |  |  |  |  |
| Duration immobile arena (s) | M | 705.0 (576.5) | 106.0 (236.8) w | 42.0 (264.5) ww |
|  | F | 684.0 (548.5) | 74.0 (147.5) ww | 42.0 (164.0) ww |
|  |  |  |  |  |
| Zone transitions white-black | M | 4.0 (12.0) | 13.0 (32.0) | 15.0 (22.5) |
|  | F | 6.0 (7.0) | 8.0 (11.5) | 17.0 (32.5) |
|  |  |  |  |  |
| Zone transitions black-white | M | 5.0 (10.5) | 13.5 (31.3) | 16.0 (23.5) |
|  | F | 7.0 (7.0) | 9.0 (12.0) | 18.0 (32.0) |

Data are presented as median (quartile range). If a fish did not visit a zone this was considered a missing value in the statistical analysis and occurrence (fish entering a zone/number of tested fish) is shown for the zones that were not visited by all fish. ^w^p<0.05, ^ww^p<0.01 compared to wild zebrafish within the respective sex; ^aa^p<0.01 compared to AB fish within the respective sex; *p<0.05 compared to females within the respective line (Mann-Whitney U-test).

**Supplementary Table 6.** Summary of statistical analyses of behavioral data from the scototaxis test in male (M) and female (F) adult offspring of wild-caught (wild), AB and *fgfr1a ^-/-^* zebrafish.

| **Zone** | **Sex** | **Wild vs AB** | **Wild vs *fgfr1a ^-/-^*** | **AB vs *fgfr1a ^-/-^*** | **KW** |
| --- | --- | --- | --- | --- | --- |
| **White compartment** |  |  |  |  |  |
| Occurrence | M | 8/9 | 8/8 | 7/9 |  |
|  | F | 8/9 | 8/9 | 9/9 |  |
|  |  |  |  |  |  |
| Latency (s) | M | n.s. | n.s. | n.s. | n.s. |
|  | F | n.s. | n.s. | n.s. | n.s. |
|  |  |  |  |  |  |
| Frequency | M | n.s. | U=8.5; p<0.05 | n.s. | H=6.0, N=23; p<0.05 |
|  | F | n.s. | n.s. | n.s. | n.s. |
|  |  |  |  |  |  |
| Duration (s) | M | n.s. | n.s. | n.s. | n.s. |
|  | F | n.s. | n.s. | n.s. | n.s. |
|  |  |  |  |  |  |
| Duration per visit (s) | M | n.s. | n.s. | n.s. | n.s. |
|  | F | n.s. | n.s. | n.s. | n.s. |
|  |  |  |  |  |  |
| Distance (cm) | M | U=10.0; p<0.05 | U=7.0; p<0.05 | n.s. | H=7.8, N=23; p<0.05 |
|  | F | U=12.0; p<0.05 | n.s. | U=8.0; p<0.01 | H=7.9, N=25; p<0.05 |
|  |  |  |  |  |  |
| Velocity (cm/s) | M | n.s. | U=8.5; p<0.05 | n.s. | H=6.9, N=23; p<0.05 |
|  | F | n.s. | n.s. | n.s. | n.s. |
|  |  |  |  |  |  |
| Duration moving (s) | M | U=8.0; p<0.05 | U=6.0; p<0.05 | n.s. | H=8.9, N=23; p<0.05 |
|  | F | U=10.0; p<0.05 | n.s. | U=6.0; p<0.01 | H=9.3, N=25; p<0.01 |
|  |  |  |  |  |  |
| Duration immobile (s) | M | n.s. | U=9.5; p<0.05 | n.s. | H=6.7, N=23; p<0.05 |
|  | F | n.s. | n.s. | n.s. | n.s. |
|  |  |  |  |  |  |
| **Black compartment** |  |  |  |  |  |
| Occurrence | M | 8/9 | 8/8 | 9/9 |  |
|  | F | 9/9 | 8/9 | 9/9 |  |
|  |  |  |  |  |  |
| Latency (s) | M | n.s. | n.s. | n.s. | n.s. |
|  | F | n.s. | n.s. | n.s. | n.s. |
|  |  |  |  |  |  |
| Frequency | M | n.s. | n.s. | n.s. | n.s. |
|  | F | n.s. | n.s. | n.s. | n.s. |
|  |  |  |  |  |  |
| Duration (s) | M | n.s. | n.s. | n.s. | n.s. |
|  | F | n.s. | n.s. | n.s. | n.s. |
|  |  |  |  |  |  |
| Duration per visit (s) | M | n.s. | n.s. | n.s. | n.s. |
|  | F | n.s. | n.s. | n.s. | n.s. |
|  |  |  |  |  |  |
| Distance (cm) | M | n.s. | n.s. | n.s. | n.s. |
|  | F | n.s. | n.s. | n.s. | n.s. |
|  |  |  |  |  |  |
| Velocity (cm/s) | M | n.s. | n.s. | n.s. | n.s. |
|  | F | U=14.0; p<0.05 | U=13.0; p<0.05 | n.s. | H=7.8, N=26; p<0.05 |
|  |  |  |  |  |  |
| Duration moving (s) | M | n.s. | n.s. | n.s. | n.s. |
|  | F | n.s. | n.s. | n.s. | n.s. |
|  |  |  |  |  |  |
| Duration immobile (s) | M | n.s. | n.s. | n.s. | n.s. |
|  | F | U=15.0; p<0.05 | U=13.0; p<0.05 | n.s. | H=6.9, N=26; p<0.05 |
|  |  |  |  |  |  |
| **Activity** |  |  |  |  |  |
| Total activity | M | n.s. | n.s. | n.s. | n.s. |
|  | F | n.s. | n.s. | n.s. | n.s. |
|  |  |  |  |  |  |
| Distance arena (cm) | M | n.s. | U=13.0; p<0.05 | n.s. | H=6.5, N=26; p<0.05 |
|  | F | n.s. | n.s. | n.s. | n.s. |
|  |  |  |  |  |  |
| Velocity arena (cm/s) | M | n.s. | U=13.0; p<0.05 | n.s. | H=6.5, N=26; p<0.05 |
|  | F | n.s. | n.s. | n.s. | n.s. |
|  |  |  |  |  |  |
| Duration moving arena (s) | M | U=12.0; p<0.05 | U=10.0; p<0.01 | n.s. | H=9.0, N=26; p<0.05 |
|  | F | U=5.0; p<0.01 | U=11.0; p<0.01 | n.s. | H=11.4, N=27; p<0.01 |
|  |  |  |  |  |  |
| Duration immobile arena (s) | M | U=12.0; p<0.05 | U=10.0; p<0.01 | n.s. | H=9.0, N=26; p<0.05 |
|  | F | U=5.0; p<0.01 | U=11.0; p<0.01 | n.s. | H=11.4, N=27; p<0.01 |
|  |  |  |  |  |  |
| Zone transitions white-black | M | n.s. | n.s. | n.s. | n.s. |
|  | F | n.s. | n.s. | n.s. | n.s. |
|  |  |  |  |  |  |
| Zone transitions black-white | M | n.s. | n.s. | n.s. | n.s. |
|  | F | n.s. | n.s. | n.s. | n.s. |

Data from the three different strains were compared within sex using a Kruskal-Wallis analysis of variance (KW) whereas the Mann-Whitney U-test was applied for pairwise comparisons between strains within sex. Values presented are Mann-Whitney U-values (U) and p-values, and Kruskal-Wallis (KW) H-value (H), total number of fish (N) and p-values. Non-significant results are indicated by n.s. If a fish did not visit a zone this was considered a missing value in the statistical analysis and occurrence (fish entering a zone/number of tested fish) is shown for the zones that were not visited by all fish.
